# Publisher Correction: Deep coverage whole genome sequences and plasma lipoprotein(a) in individuals of European and African ancestries

**DOI:** 10.1038/s41467-018-05975-y

**Published:** 2018-08-23

**Authors:** Seyedeh M. Zekavat, Sanni Ruotsalainen, Robert E. Handsaker, Maris Alver, Jonathan Bloom, Timothy Poterba, Cotton Seed, Jason Ernst, Mark Chaffin, Jesse Engreitz, Gina M. Peloso, Ani Manichaikul, Chaojie Yang, Kathleen A. Ryan, Mao Fu, W. Craig Johnson, Michael Tsai, Matthew Budoff, Ramachandran S. Vasan, L. Adrienne Cupples, Jerome I. Rotter, Stephen S. Rich, Wendy Post, Braxton D. Mitchell, Adolfo Correa, Andres Metspalu, James G. Wilson, Veikko Salomaa, Manolis Kellis, Mark J. Daly, Benjamin M. Neale, Steven McCarroll, Ida Surakka, Tonu Esko, Andrea Ganna, Samuli Ripatti, Sekar Kathiresan, Pradeep Natarajan, NHLBI TOPMed Lipids Working Group, Namiko Abe, Goncalo Abecasis, Christine Albert, Nicholette (Nichole) Palmer Allred, Laura Almasy, Alvaro Alonso, Seth Ament, Peter Anderson, Pramod Anugu, Deborah Applebaum-Bowden, Dan Arking, Donna K Arnett, Allison Ashley-Koch, Stella Aslibekyan, Tim Assimes, Paul Auer, Dimitrios Avramopoulos, John Barnard, Kathleen Barnes, R. Graham Barr, Emily Barron-Casella, Terri Beaty, Diane Becker, Lewis Becker, Rebecca Beer, Ferdouse Begum, Amber Beitelshees, Emelia Benjamin, Marcos Bezerra, Larry Bielak, Joshua Bis, Thomas Blackwell, John Blangero, Eric Boerwinkle, Ingrid Borecki, Russell Bowler, Jennifer Brody, Ulrich Broeckel, Jai Broome, Karen Bunting, Esteban Burchard, Jonathan Cardwell, Cara Carty, Richard Casaburi, James Casella, Christy Chang, Daniel Chasman, Sameer Chavan, Bo-Juen Chen, Wei-Min Chen, Yii-Der Ida Chen, Michael Cho, Seung Hoan Choi, Lee-Ming Chuang, Mina Chung, Elaine Cornell, Carolyn Crandall, James Crapo, Joanne Curran, Jeffrey Curtis, Brian Custer, Coleen Damcott, Dawood Darbar, Sayantan Das, Sean David, Colleen Davis, Michelle Daya, Mariza de Andrade, Michael DeBaun, Ranjan Deka, Dawn DeMeo, Scott Devine, Ron Do, Qing Duan, Ravi Duggirala, Peter Durda, Susan Dutcher, Charles Eaton, Lynette Ekunwe, Patrick Ellinor, Leslie Emery, Charles Farber, Leanna Farnam, Tasha Fingerlin, Matthew Flickinger, Myriam Fornage, Nora Franceschini, Stephanie M. Fullerton, Lucinda Fulton, Stacey Gabriel, Weiniu Gan, Yan Gao, Margery Gass, Bruce Gelb, Xiaoqi (Priscilla) Geng, Soren Germer, Chris Gignoux, Mark Gladwin, David Glahn, Stephanie Gogarten, Da-Wei Gong, Harald Goring, C. Charles Gu, Yue Guan, Xiuqing Guo, Jeff Haessler, Michael Hall, Daniel Harris, Nicola Hawley, Jiang He, Ben Heavner, Susan Heckbert, Ryan Hernandez, David Herrington, Craig Hersh, Bertha Hidalgo, James Hixson, John Hokanson, Elliott Hong, Karin Hoth, Chao (Agnes) Hsiung, Haley Huston, Chii Min Hwu, Marguerite Ryan Irvin, Rebecca Jackson, Deepti Jain, Cashell Jaquish, Min A Jhun, Jill Johnsen, Andrew Johnson, Rich Johnston, Kimberly Jones, Hyun Min Kang, Robert Kaplan, Sharon Kardia, Laura Kaufman, Shannon Kelly, Eimear Kenny, Michael Kessler, Alyna Khan, Greg Kinney, Barbara Konkle, Charles Kooperberg, Holly Kramer, Stephanie Krauter, Christoph Lange, Ethan Lange, Leslie Lange, Cathy Laurie, Cecelia Laurie, Meryl LeBoff, Seunggeun Shawn Lee, Wen-Jane Lee, Jonathon LeFaive, David Levine, Dan Levy, Joshua Lewis, Yun Li, Honghuang Lin, Keng Han Lin, Simin Liu, Yongmei Liu, Ruth Loos, Steven Lubitz, Kathryn Lunetta, James Luo, Michael Mahaney, Barry Make, JoAnn Manson, Lauren Margolin, Lisa Martin, Susan Mathai, Rasika Mathias, Patrick McArdle, Merry-Lynn McDonald, Sean McFarland, Stephen McGarvey, Hao Mei, Deborah A Meyers, Julie Mikulla, Nancy Min, Mollie Minear, Ryan L Minster, May E. Montasser, Solomon Musani, Stanford Mwasongwe, Josyf C Mychaleckyj, Girish Nadkarni, Rakhi Naik, Sergei Nekhai, Deborah Nickerson, Kari North, Jeff O’Connell, Tim O’Connor, Heather Ochs-Balcom, James Pankow, George Papanicolaou, Margaret Parker, Afshin Parsa, Sara Penchev, Juan Manuel Peralta, Marco Perez, James Perry, Ulrike Peters, Patricia Peyser, Larry Phillips, Sam Phillips, Toni Pollin, Julia Powers Becker, Meher Preethi Boorgula, Michael Preuss, Dmitry Prokopenko, Bruce Psaty, Pankaj Qasba, Dandi Qiao, Zhaohui Qin, Nicholas Rafaels, Laura Raffield, D. C. Rao, Laura Rasmussen-Torvik, Aakrosh Ratan, Susan Redline, Robert Reed, Elizabeth Regan, Alex Reiner, Ken Rice, Dan Roden, Carolina Roselli, Ingo Ruczinski, Pamela Russell, Sarah Ruuska, Phuwanat Sakornsakolpat, Shabnam Salimi, Steven Salzberg, Kevin Sandow, Vijay Sankaran, Christopher Scheller, Ellen Schmidt, Karen Schwander, David Schwartz, Frank Sciurba, Christine Seidman, Vivien Sheehan, Amol Shetty, Aniket Shetty, Wayne Hui-Heng Sheu, M. Benjamin Shoemaker, Brian Silver, Edwin Silverman, Jennifer Smith, Josh Smith, Nicholas Smith, Tanja Smith, Sylvia Smoller, Beverly Snively, Tamar Sofer, Nona Sotoodehnia, Adrienne Stilp, Elizabeth Streeten, Yun Ju Sung, Jody Sylvia, Adam Szpiro, Carole Sztalryd, Daniel Taliun, Hua Tang, Margaret Taub, Kent Taylor, Simeon Taylor, Marilyn Telen, Timothy A. Thornton, Lesley Tinker, David Tirschwell, Hemant Tiwari, Russell Tracy, Dhananjay Vaidya, Peter VandeHaar, Scott Vrieze, Tarik Walker, Robert Wallace, Avram Walts, Emily Wan, Fei Fei Wang, Karol Watson, Daniel E. Weeks, Bruce Weir, Scott Weiss, Lu-Chen Weng, Cristen Willer, Kayleen Williams, L. Keoki Williams, Carla Wilson, Quenna Wong, Huichun Xu, Lisa Yanek, Ivana Yang, Rongze Yang, Norann Zaghloul, Yingze Zhang, Snow Xueyan Zhao, Wei Zhao, Xiuwen Zheng, Degui Zhi, Xiang Zhou, Michael Zody, Sebastian Zoellner

**Affiliations:** 1 Program in Medical and Population Genetics, Broad Institute of MIT and Harvard, Cambridge, MA 02142 USA; 2 Yale School of Medicine, New Haven, CT 06510 USA; 3 Department of Computational Biology & Bioinformatics, Yale University, New Haven, CT 06510 USA; 4 Institute for Molecular Medicine, University of Helsinki, Helsinki, Finland; 5 Stanley Center for Psychiatric Research, Broad Institute of MIT and Harvard, Cambridge, MA 02142 USA; 6 Department of Genetics, Harvard Medical School, Boston, MA 02115 USA; 7 Analytic and Translational Genetics Unit, Boston, MA 02142 USA; 8 Department of Biotechnology, Institute of Molecular and Cell Biology, University of Tartu, Tartu, Estonia; 9 Estonian Genome Center, Tallinn, Estonia; 10 Department of Biological Chemistry, University of California, Los Angeles, Los Angeles, CA 90095 USA; 11 Department of Biostatistics, Boston University School of Public Health, Boston, MA 02118 USA; 12 Center for Public Health Genomics, University of Virginia, Charlottesville, VA 22904 USA; 13 Program in Personalized and Genomic Medicine, Division of Endocrinology, Diabetes & Nutrition, Department of Medicine, University of Maryland School of Medicine, Baltimore, MD 21201 USA; 14 Department of Biostatistics, School of Public Health and Community Medicine, University of Washington, Seattle, WA 98195 USA; 15 Department of Laboratory Medicine and Pathology, University of Minnesota, Minneapolis, MN 55455 USA; 16 Division of Cardiology, Harbor-UCLA Medical Center, Los Angeles Biomedical Research Institute, Los Angeles, CA 90509 USA; 17 NHLBI Framingham Heart Study, Framingham, MA 20892 USA; 18 Sections of Preventive medicine and Epidemiology, and cardiovascular medicine, Departments of Medicine and Epidemiology, Boston university Schools of Medicine and Public health, Boston, MA 02118 USA; 19 Departments of Pediatrics and Medicine, The Institute for Translational Genomics and Population Sciences, Los Angeles Biomedical Research Institute, Harbor-UCLA Medical Center, Torrance, CA 90509 USA; 20 Division of Cardiology, Department of Medicine, Johns Hopkins University School of Medicine, Baltimore, MD 21205 USA; 21 Department of Medicine, University of Maryland School of Medicine, Baltimore, MD 21201 USA; 22 Department of Medicine, University of Mississippi Medical Center, Jackson, MS 39216 USA; 23 National Institute for Health and Welfare, Helsinki, Finland; 24 Computer Science and Artificial Intelligence Lab, Massachusetts Institute of Technology, 32 Vassar St, Cambridge, MA 02139 USA; 25 Department of Public Health, Faculty of Medicine, University of Helsinki, Helsinki, Finland; 26 Department of Medicine, Harvard Medical School, Boston, MA 02115 USA; 27 Center for Genomic Medicine, Massachusetts General Hospital, Boston, MA 02114 USA; 28 Cardiovascular Research Center, Massachusetts General Hospital, Boston, MA 02114 USA; 29 New York Genome Center, New York, NY 10013 USA; 30 University of Michigan, Ann Arbor, MI 48109 USA; 31 Massachusetts General Hospital, Boston, MA 02114 USA; 32 Wake Forest Baptist Health, Winston-Salem, NC 27157 USA; 33 Children’s Hospital of Philadelphia, University of Pennsylvania, Philadelphia, PA 19104 USA; 34 University of Pennsylvania, Philadelphia, PA 19104 USA; 35 Emory University, Atlanta, GA 30322 USA; 36 University of Maryland, Baltimore, MD 21201 USA; 37 University of Washington, Seattle, WA 98195 USA; 38 University of Mississippi, Jackson, MS 38677 USA; 39 National Institutes of Health, Bethesda, MD 20892 USA; 40 Johns Hopkins University, Baltimore, MD 21218 USA; 41 University of Kentucky, Lexington, KY 40506 USA; 42 Duke University, Durham, NC 27708 USA; 43 University of Alabama, Birmingham, AL 35487 USA; 44 Stanford University, Stanford, CA 94305 USA; 45 University of Wisconsin Milwaukee, Milwaukee, WI 53211 USA; 46 Cleveland Clinic, Cleveland, OH 44195 USA; 47 University of Colorado, Denver, CO USA 80204; 48 Columbia University, New York, NY 10027 USA; 49 Boston University, Boston, MA 02215 USA; 50 Fundação de Hematologia e Hemoterapia de Pernambuco - Hemope, Recife, 52011-000 Brazil; 51 University of Texas Rio Grande Valley School of Medicine, Brownsville, TX 78520 USA; 52 University of Texas Health, Houston, TX 77225 USA; 53 National Jewish Health, Denver, CO 80206 USA; 54 Medical College of Wisconsin, Milwaukee, WI 53226 USA; 55 University of California, San Francisco, San Francisco, CA 94143 USA; 56 Women’s Health Initiative, Seattle, WA 98109 USA; 57 University of California, Los Angeles, Los Angeles, CA 90095 USA; 58 Brigham & Women’s Hospital, Boston, MA 02115 USA; 59 University of Virginia, Charlottesville, VA 22903 USA; 60 Los Angeles Biomedical Research Institute, Los Angeles, CA 90502 USA; 61 The Broad Institute, Cambridge, MA 02142 USA; 62 National Taiwan University, Taipei, 10617 Taiwan; 63 University of Vermont, Burlington, VT 05405 USA; 64 Blood Systems Research Institute UCSF, San Francisco, CA 94118 USA; 65 University of Illinois at Chicago, Chicago, IL 60607 USA; 66 Mayo Clinic, Rochester, MN 55905 USA; 67 Vanderbilt University, Nashville, TN 37235 USA; 68 University of Cincinnati, Cincinnati, OH 45220 USA; 69 Icahn School of Medicine at Mount Sinai, New York, NY 10029 USA; 70 University of North Carolina, Chapel Hill, NC 27599 USA; 71 University of Texas Rio Grande Valley School of Medicine, Edinburg, TX 78539 USA; 72 Washington University in St Louis, St Louis, MO 63130 USA; 73 Brown University, Providence, RI 02912 USA; 74 Fred Hutchinson Cancer Research Center, Seattle, WA 98109 USA; 75 University of Pittsburgh, Pittsburgh, PA 15260 USA; 76 Yale University, New Haven, CT 06520 USA; 77 University of Texas Rio Grande Valley School of Medicine, San Antonio, TX 78229 USA; 78 Tulane University, New Orleans, LA 70118 USA; 79 University of Iowa, Iowa City, IA 52242 USA; 80 National Health Research Institute Taiwan, Zhunan Township, 350 Taiwan; 81 Blood Works Northwest, Seattle, WA 98105 USA; 82 Taichung Veterans General Hospital Taiwan, Taichung City, 407 Taiwan; 83 Ohio State University Wexner Medical Center, Columbus, OH 43210 USA; 84 NIH National Heart, Lung, and Blood Institute, Bethesda, MD 98106 USA; 85 Albert Einstein College of Medicine, New York, NY 20892 USA; 86 Loyola University, Maywood, IL 10461 USA; 87 Harvard School of Public Health, Boston, MA 98104 USA; 88 George Washington University, Washington, 60153 USA; 89 Harvard University, Cambridge, MA 02115 USA; 90 University of Arizona, Tucson, AZ 20052 USA; 91 Howard University, Washington, 02138 USA; 92 University at Buffalo, Buffalo, NY 85721 USA; 93 University of Minnesota, Minneapolis, MN 20059 USA; 94 Northwestern University, Chicago, IL 14260 USA; 95 Harvard Medical School, Boston, MA 55455 USA; 96 Baylor College of Medicine, Houston, TX 60208 USA; 97 UMass Memorial Medical Center, Worcester, MA 98107 USA; 98 Baylor College of Medicine, Ann Arbor, MI 02115 USA; 99 University of Colorado at Boulder, Boulder, CO 77030 USA; 100 Henry Ford Health System, Detroit, MI 01655 USA

Correction to: *Nature Communications*; 10.1038/s41467-018-04668-w, published online 4 July 2018

