# Supplementary material for: Deep coverage whole genome sequences and plasma lipoprotein(a) in individuals of European and African ancestries
Source: Nat Commun. 2018 Jul 4;9:2606. doi: 10.1038/s41467-018-04668-w (PMC6031652; doi:10.1038/s41467-018-04668-w)
Supplement: Supplementary file 1 — Supplementary Information [file 41467_2018_4668_MOESM1_ESM.pdf]

Supplementary Information

Deep coverage whole genome sequences and plasma lipoprotein(a) in individuals of European and African ancestries

Zekavat et al.

Supplementary Note 1: Main Study Participants

Jackson Heart Study (JHS)

The JHS is a community-based cohort among 5,306 African Americans in the Jackson, Mississippi metropolitan area[1] whose data and biologic materials have been collected during a baseline examination (2000-2004) and two follow-up examinations (2005-2008 and 2009-2013), with ongoing surveillance for hospitalizations for myocardial infarction, coronary heart disease (CHD) and stroke, hospitalizations for heart failure (since 2005), and CHD and overall mortality. The age at enrollment for the unrelated individuals was 35-84 years; a nested family cohort of 1,498 members of 264 families included related individuals >21 years old. High prevalence of diabetes, hypertension, obesity, and related disorders is present in this population.

FINRISK (National FINRISK Study)

FINRISK was a population-based cross-sectional survey designed to study the prevalence of cardiovascular risk factors in Finland[2]. FINRISK surveys were conducted in 1992, 1997, 2002, and 2007 from men and women aged 25-74 who underwent a questionnaire and clinical examination during which blood samples were drawn and used towards genotyping and metabolic profiling. Linkage to national registers of cardiovascular and other health outcomes was available for defining prevalent and incident clinical cases.

EGCUT (Estonian Genome Center of University of Tartu)

The Estonian cohort is from the population-based biobank of the Estonian Genome Project of University of Tartu (EGCUT)[3]. The project is conducted according to the Estonian Gene Research Act, with over 51,515 participants aged >18 who have signed the broad informed consent. Subjects are recruited by randomly selected general practitioners (GP). At their doctor's office, each participant underwent a 1-2 hour computer-assisted personal interview which included personal data, genealogical data, educational and occupational history, and lifestyle data. Venous blood samples were collected for DNA and plasma isolation and sent to the Broad Institute for whole genome sequencing and Atherotech for the VAP blood lipid panel, respectively.

Supplementary Note 2: Additional Participants Used in Subclinical Atherosclerosis Instrumental Variable Analyses

MESA (Multi-ethnic Study of Atherosclerosis)

The Multi-Ethnic Study of Atherosclerosis[4] is a population-based investigation of subclinical cardiovascular disease and its progression among a total of 6,814 individuals, aged 45 to 84 years, who were recruited from six US communities (Baltimore City and County, MD; Chicago, IL; Forsyth County, NC; Los Angeles County, CA; New York, NY; and St. Paul, MN) between July 2000 and August 2002. Participants were excluded if they had physician-diagnosed

cardiovascular disease prior to enrollment, including angina, myocardial infarction, heart failure, stroke or TIA, resuscitated cardiac arrest or a cardiovascular intervention (e.g., CABG, angioplasty, valve replacement, or pacemaker/defibrillator placement). Lp(a) mass concentrations were measured using a turbidimetric immunoassay[5]. Only European-American and African-American individuals were used in subclinical atherosclerosis analyses.

FHS (Framingham Heart Study)

The FHS is a three-generation prospective cohort that has been described in detail previously[6]. Individuals were initially recruited in 1948 in Framingham, USA to evaluate cardiovascular disease risk factors. The second generation cohort (5,124 offspring of the original cohort) was recruited between 1971 and 1975. The third generation cohort (4,095 grandchildren of the original cohort) was collected between 2002 and 2005. Lp(a)-C levels were measured in FHS offspring participants during the 3rd examination cycle (1991-1995) using ELISA (enzyme-linked immunosorbent assay)[7].

OOA (Old Order Amish)

The Old Order Amish individuals included in this study were participants of several ongoing studies of cardiovascular health carried out at the University of Maryland among relatively healthy volunteers from the Old Order Amish community of Lancaster County, PA. Lp(a)-C measurements were made with Vertical Auto Profile-II (VAP-II) method (Atherotech, Birmingham, AL, USA)[8].

Supplementary Note 3: Imputation of KIV2-CN using variants from the Illumina OmniQuad genotyping array

To further explore the model's performance using variants present in a conventional genotyping array platform, we determined the overlap between the 61 variants and variants within the Illumina OmniQuad genotyping array using SNAP (http://archive.broadinstitute.org/mpg/snap/ldsearch.php). 6 of the 61variants (including the top two important variants) were either present or had proxies (with LD $r^2 > 0.8$) available in the OmniQuad array. Using these variants, we re-computed LASSO coefficients (listed below) using the same methodology as previously described and found the predictive performance to be lower than the 61-variant model, with Pearson coefficient of 0.62 between estimated and genotyped KIV2-CN and explaining 38% of variation in KIV2-CN.

Variant (hg19 chr.pos.ref.alt)	*rsID*	*OmniQuad Variant in LD*	*LASSO Coefficient*
6.160910517.T.A	rs12214416	rs12214416 (r^2 = 1)	1.62
6.160919223.T.C	rs4129086	rs4129086 (r^2 = 1)	0.91
6.161010118.A.G	rs10455872	rs10455872 (r^2 = 1)	-9.20
6.161068320.C.G	rs4708876	rs7770628 (r^2 = 0.87)	-2.91
6.161068607.T.C	rs12526465	rs12526465 (r^2 = 1)	-8.17

6.161233297.C.T	rs117774213	rs9458173 ($r^2 = 1$)	10.41
(Intercept)	-	-	49.48

Supplementary Note 4: Rare variant association analysis grouping schemes

Coding

Two coding RVAS tests were performed using variants with MAF < 1% grouped by gene: 1) loss-of-function (defined as most severe canonical consequence annotated as frameshift, transcript ablation, splice acceptor, splice donor, stop gained or start lost by VEP[9]) or missense deleterious variants (by MetaSVM[10]), and 2) non-synonymous variants (defined as the most severe canonical consequence annotated as missense, stop-gained, stop-lost, or start-lost by VEP)

Non-Coding, Sliding Window

We performed a "sliding window" approach aggregating 3kb (overlapping by 1.5kb) windows and considering rare variants occurring within adult liver enhancer or promoter elements at strong DNase I hypersensitivity sites.

Non-Coding, By Distance

For non-coding tests, we attempted to link rare non-coding variants with genes for association testing using regulatory annotations for adult liver as previously described under "Annotations". Prior studies have shown that approximately 80% of cis-eQTLs fall within 100kb of TSS[11]. To increase likelihood that of mapping regulatory variants to the nearest gene, we were more restrictive and included variants overlapping promoter sequences +/- 5kb and enhancer sequences +/- 20kb of TSS at strong DNase I hypersensitivity sites.

Non-Coding, By Expression

We also aggregated rare non-coding variants within chromatin state defined enhancers linked to gene by gene expression using data from the Roadmap Epigenomics project[12] and the method presented previously[13] with a few small modifications (methods described in Liu Y et al. Genome Biology[14]). This method predicts links using chromatin state information, position of the enhancer relative to the TSS, and the correlation of multiple chromatin marks with gene expression across cell types. Here we used the correlation with gene expression of the signal of five chromatin marks: H3K27ac, H3K9ac, H3K4me1, H3K4me2, and DNaseI hypersensitivity. The gene expression data was the RPKM expression data for protein coding exons across 56 reference epigenomes from the Roadmap Epigenomics project (available in the file 57epigenomes.RPKM.pc from http://compbio.mit.edu/roadmap; Universal Human Reference was excluded). The chromatin mark signal was the $-\log_{10}(P)$ tracks averaged to a 200-bp

resolution. As input to our code we used the version of those tracks first averaged at 25-bp resolution using the 'Convert' command of ChromImpute[15]. In computing correlation between a specific chromatin mark signal and gene expression we used the Pearson correlation and omitted from the calculation samples lacking both chromatin mark signal and gene expression data. We made predictions separately for each of the 127 reference epigenomes and locations assigned to chromatin states, 6_EnhG, 7_Enh, and 12_EnhBiv, of the 15-state core 5-marks ChromHMM model[12,16]. We restricted our predictions to chromatin state assignments on chr1-22 and chrX. We considered linking 200-bp bins within 1MB of a TSS of each gene as annotated in the file Ensembl_v65.Gencode_v10.ENSG.gene_info available from http://compbio.mit.edu/roadmap.[12] If a gene had multiple TSS, then we only used the outermost TSS.

The method for linking is based on determining for each combination of cell type, chromatin state, and position relative to the TSS the estimated probability the set of correlations we observed would come from the actual data compared to randomized data. To this end we created a training set of actual observed correlations (positive examples) and correlations computed after randomizing which gene expression values were assigned to which genes (negative examples) separately for each combination of cell type, chromatin state, and position relative to the TSS. Each entry in the training set has five features corresponding to correlations for each of the considered chromatin marks. There is a positive and a corresponding negative entry for each instance of the specified chromatin state in the specified cell type at the specified position relative to the TSS or within 5kb of it (for smoothing purposes). We trained a logistic regression classifier to discriminate actual correlations with randomized correlations. We used the logistic regression library implemented in the Weka package version 3.7.3[17] with the regularization parameter set to 1. For considering linking a specific instance of a chromatin state assignment in a specific cell type and position relative to the TSS of a gene we applied the corresponding classifier. Let p denote the probability the classifier gives of being in the positive class of the actual observed correlations. We retained those links for which p/(1-p) was greater than or equal to 2.5. The method we used here is implemented in the code LinkingRM.java at the following page: https://github.com/dnaase/Bis-tools/tree/master/recombination_valley_paper. Predictions are available at http://www.biolchem.ucla.edu/labs/ernst/roadmaplinking/. For the analyses presented here we combined links for the enhancer states: 6_EnhG, 7_Enh, and 12_EnhBiv.

Supplementary Note 5: Phenotypes used in Mendelian randomization

Incident events

Incident events were defined as follows in the FINRISK cohorts. The ICD-10 and ICD-9 below refer to the Finnish versions of the ICD-codes.

(**1) Myocardial infarction MI)** was defined as either cause of death or hospital discharge with ICD codes I21–I22 (ICD-10) or 410 (ICD-9).

2) Coronary heart disease (CHD) was defined as underlying or direct cause of death with ICD codes I20–I25, I46, R96 or R98 (ICD-10) or 410–414 or 798 (ICD-9), or as the main diagnosis at hospital discharge with ICD codes I200, I21–I22 (ICD-10) or 410, 4110 (ICD-9) or as coronary bypass surgery or coronary angioplasty at hospital discharge or identified from the specific country-wide register of invasive cardiac procedures. The definition of CHD includes all MI cases.

3) Stroke (excluding subarachnoid hemorrhage) was defined either as the underlying or direct cause of death or as the main or side diagnosis at hospital discharge with ICD codes I61, I63, I64 except I63.6 (ICD-10) or 431, 4330A, 4331A, 4339A, 4340A, 4341A, 4349A, 436 (ICD-9). Of the individuals analyzed, 80% had ischemic stroke, 13% had hemorrhagic stroke, and 7% had both.

4) Cardiovascular disease (CVD): Either MI, CHD or stroke.

5) Acute Coronary Syndrome was defined as either cause of death or hospital discharge with ICD codes I200, I21, or I22 (ICD-10), or 410 or 4110 (ICD-8/9) (hospital discharge) or I20-I25 (ICD-10) or 410-414 (ICD-8/9) (cause of death).

6) Cancer (any kind): was defined as either cause of death or hospital discharge with ICD codes C00-C43, C45-C97 (ICD-10) 140-172, 174-208 (ICD-8/9).

7) Dementia: Dementia in Alzheimer disease or other diseases, vascular or unspecified dementia, was defined as either cause of death or hospital discharge with ICD codes F00, F01, F02, F03, G30 (ICD-10); 3310, 4378A (ICD-9), 290 (ICD-8/9) or at least three prescription medicine purchases with ATC class N06D, or as the specially reimbursed medication for dementia.

8) Heart failure: Heart failure (congestive), was defined as either cause of death or hospital discharge with ICD codes I50, I110, I130, I132 (ICD-10); 4029B, 4148, 428 (ICD-9), 42700, 42710, 428 (ICD-8) or at least three prescription medicine purchases with ATC classes C03CA01, C03EB01, or as the specially reimbursed medication for heart failure.

9) Diabetes: Diabetes mellitus, was defined as underlying or direct cause of death or as the main or side diagnosis at hospital discharge with ICD codes E10-E14 (ICD-10) / 250 (ICD-8/9) with ICD codes E10, E11, E14 (ICD-10); 250 (ICD-8/9) or at least three prescription medicine purchases with ATC classes A10, A10A and A10B, or as the specially reimbursed medication for diabetes.

Note: only 29 incident cases with chronic kidney failure and Lp(a) phenotype were available, out of a total of 69 incident cases; thus, due to lack of power, analysis using this phenotype was not performed.

A non-fatal event prior to or at the clinical examination date was considered prevalent. An event during the follow-up in persons with no history of prior events was considered incident.

Sub-clinical atherosclerosis phenotypes

JHS

Coronary artery calcium (CAC) and abdominal aortic calcium (AAC) were obtained in JHS as previously described[18]. Briefly, computed tomography (CT) imaging of the torso were obtained by multi-detector CT (GE Healthcare Lightspeed 16 Pro, Wakeshau, Wisconsin) during Exam 2 at the Jackson Medical Mall. CAC and AAC were quantified utilizing Agatston scoring, modified to account for slice thickness; the reproducibility in scoring was 0.99.

MESA

CAC was measured with either electron-beam CT or multi-detector CT, at one of three field centers. Each participant was scanned twice consecutively, and these scans were read independently at a centralized reading center. The methodology for acquisition and interpretation of the scans has been documented previously[19,20]. CAC was quantified utilizing the Agatston scoring method.

OOA

CAC and AAC were obtained via electron-beam CT scans and quantified using the Agatston scoring method as previously described[21].

FHS

Participants of the Framingham Offspring and Third Generation Cohorts underwent multi-detector CT scanning from 2002 to 2005 with repeat scans occurring from 2008 to 2010. CAC and AAC were quantified using the Agatston scoring method as previously described[22].

Supplementary Note 6: Acknowledgements

Whole genome sequencing (WGS) for the Trans-Omics in Precision Medicine (TOPMed) program was supported by the National Heart, Lung and Blood Institute (NHLBI). WGS for "NHLBI TOPMed: The Jackson Heart Study" (phs000964.v1.p1) was performed at the University of Washington Northwest Genomics Center (HHSN268201100037C). Centralized read mapping and genotype calling, along with variant quality metrics and filtering were provided by the TOPMed Informatics Research Center (3R01HL-117626-02S1). The Jackson Heart Study (JHS) is supported and conducted in collaboration with Jackson State University (HHSN268201300049C and HHSN268201300050C), Tougaloo College (HHSN268201300048C), and the University of Mississippi Medical Center (HHSN268201300046C and HHSN268201300047C) contracts from the National Heart, Lung, and Blood Institute (NHLBI) and the National Institute for Minority Health and Health Disparities (NIMHD). WGS for "NHLBI TOPMed: Multi-Ethnic Study of Atherosclerosis

(MESA)" (phs001416.v1.p1) was performed at the Broad Institute of MIT and Harvard (3U54HG003067-13S1). MESA and the MESA SHARe project are conducted and supported by the National Heart, Lung, and Blood Institute (NHLBI) in collaboration with MESA investigators. Support for MESA is provided by contracts HHSN268201500003I, N01-HC-95159, N01-HC-95160, N01-HC-95161, N01-HC-95162, N01-HC-95163, N01-HC-95164, N01-HC-95165, N01-HC-95166, N01-HC-95167, N01-HC-95168, N01-HC-95169, UL1-TR-000040, UL1-TR-001079, UL1-TR-001420. The provision of genotyping data was supported in part by the National Center for Advancing Translational Sciences, CTSI grant UL1TR001881, and the National Institute of Diabetes and Digestive and Kidney Disease Diabetes Research Center (DRC) grant DK063491 to the Southern California Diabetes Endocrinology Research Center. Phenotype harmonization, data management, sample-identity QC, and general study coordination, were provided by the TOPMed Data Coordinating Center (3R01HL-120393-02S1). We gratefully acknowledge the studies and participants who provided biological samples and data for TOPMed, Estonia, and FINRISK cohorts. The authors also wish to thank the staffs and participants of the JHS, Estonia, and FINRISK cohorts. S.K. is supported by an Ofer and Shelly Nemirovsky Research Scholar Award from Massachusetts General Hospital, RO1 HL127564 from the National Heart, Lung, and Blood Institute, and UM HG008895 from the National Human Genome Research Institute. T.E. and A.M. are funded by Estonian Research Council Grant IUT20-60 and PUT1660, EU H2020 grant 692145, and European Union through the European Regional Development Fund (Project No. 2014-2020.4.01.15-0012) GENTRANSMED. J.G.W. is supported by U54GM115428 from the National Institute of General Medical Sciences. I.S. is supported by the Academy of Finland (298149). V.S is supported by the Finnish Foundation for Cardiovascular Research. The Amish studies were supported by NIH grants R01 HL69313, R01 HL088119, R01 HL121007, AHA17GRNT33440151, and P30 DK072488. The Framingham Heart Study has been supported by contracts N01-HC-25195 and HHSN268201500001I and grant R01 HL092577. The Framingham Heart Study thanks the study participants and the multitude of investigators who over its 70-year history continue to contribute so much to further our knowledge of heart, lung, blood and sleep disorders and associated traits. S.M.Z is supported by the National Institutes of Health's Medical Scientist Training Program at the Yale School of Medicine and the Paul & Daisy Soros Fellowship. The views expressed in this manuscript are those of the authors and do not necessarily represent the views of the National Heart, Lung, and Blood Institute; the National Institutes of Health; or the U.S. Department of Health and Human Services.

References:

1. Taylor, H.A., Jr. *et al*. Toward resolution of cardiovascular health disparities in African Americans: design and methods of the Jackson Heart Study. *Ethn Dis* **15**, S6-4-17 (2005).
2. Vartiainen, E. *et al*. Cardiovascular risk factor changes in Finland, 1972-1997. *Int J Epidemiol* **29**, 49-56 (2000).
3. Nelis, M. *et al*. Genetic structure of Europeans: a view from the North-East. *PLoS One* **4**, e5472 (2009).
4. Bild, D.E. *et al*. Multi-Ethnic Study of Atherosclerosis: objectives and design. *Am J Epidemiol* **156**, 871-81 (2002).
5. Guan, W. *et al*. Race is a key variable in assigning lipoprotein(a) cutoff values for coronary heart disease risk assessment: the Multi-Ethnic Study of Atherosclerosis. *Arterioscler Thromb Vasc Biol* **35**, 996-1001 (2015).
6. Kannel, W.B., Dawber, T.R., Kagan, A., Revotskie, N. & Stokes, J., 3rd. Factors of risk in the development of coronary heart disease--six year follow-up experience. The Framingham Study. *Ann Intern Med* **55**, 33-50 (1961).
7. Thanassoulis, G. *et al*. Genetic associations with valvular calcification and aortic stenosis. *N Engl J Med* **368**, 503-12 (2013).
8. Lu, W. *et al*. Evidence for several independent genetic variants affecting lipoprotein (a) cholesterol levels. *Hum Mol Genet* **24**, 2390-400 (2015).
9. McLaren, W. *et al*. Deriving the consequences of genomic variants with the Ensembl API and SNP Effect Predictor. *Bioinformatics* **26**, 2069-70 (2010).
10. Dong, C. *et al*. Comparison and integration of deleteriousness prediction methods for nonsynonymous SNVs in whole exome sequencing studies. *Hum Mol Genet* **24**, 2125-37 (2015).
11. Consortium, G.T. Human genomics. The Genotype-Tissue Expression (GTEx) pilot analysis: multitissue gene regulation in humans. *Science* **348**, 648-60 (2015).
12. Zhou, X. *et al*. Epigenomic annotation of genetic variants using the Roadmap Epigenome Browser. *Nat Biotechnol* **33**, 345-6 (2015).
13. Ernst, J. *et al*. Mapping and analysis of chromatin state dynamics in nine human cell types. *Nature* **473**, 43-9 (2011).
14. Liu, Y., Sarkar, A., Kheradpour, P., Ernst, J. & Kellis, M. Evidence of reduced recombination rate in human regulatory domains. *Genome Biol* **18**, 193 (2017).
15. Ernst, J. & Kellis, M. Large-scale imputation of epigenomic datasets for systematic annotation of diverse human tissues. *Nat Biotechnol* **33**, 364-76 (2015).
16. Ernst, J. & Kellis, M. ChromHMM: automating chromatin-state discovery and characterization. *Nat Methods* **9**, 215-6 (2012).
17. Frank, E., Hall, M., Trigg, L., Holmes, G. & Witten, I.H. Data mining in bioinformatics using Weka. *Bioinformatics* **20**, 2479-81 (2004).
18. Tullos, B.W. *et al*. Ankle-brachial index (ABI), abdominal aortic calcification (AAC), and coronary artery calcification (CAC): the Jackson heart study. *Int J Cardiovasc Imaging* **29**, 891-7 (2013).
19. McClelland, R.L., Chung, H., Detrano, R., Post, W. & Kronmal, R.A. Distribution of coronary artery calcium by race, gender, and age: results from the Multi-Ethnic Study of Atherosclerosis (MESA). *Circulation* **113**, 30-7 (2006).

20. Carr, J.J. *et al*. Calcified coronary artery plaque measurement with cardiac CT in population-based studies: standardized protocol of Multi-Ethnic Study of Atherosclerosis (MESA) and Coronary Artery Risk Development in Young Adults (CARDIA) study. *Radiology* **234**, 35-43 (2005).
21. Post, W. *et al*. Determinants of coronary artery and aortic calcification in the Old Order Amish. *Circulation* **115**, 717-24 (2007).
22. Onuma, O.K. *et al*. Relation of Risk Factors and Abdominal Aortic Calcium to Progression of Coronary Artery Calcium (from the Framingham Heart Study). *Am J Cardiol* **119**, 1584-1589 (2017).

Supplementary Table 1: Sample-level quality control of the whole-genome sequencing cohorts

	JHS	FIN	EST	ALL
Contamination*	1	8	12	21
Chimeras > 5%	0	4	0	4
GC dropout > 4	0	1	0	1
Raw coverage†	0	0	4	4
Indeterminate genotypic sex‡	0	2	0	2
Duplicates / monozygotic twins§	2	0	0	2
Expected population outliers by PCA	14	0	0	14
Variant metric count outliers	2	0	0	2
Array-sequencing genotype concordance < 0.95	0	0	10	10
TOTAL FILTERED	19	15	26	

† Raw coverage threshold for JHS was <30X but was <19X for the combined Finland and Estonia callset.

‡ Chromosome X F inbreeding coefficient 0.5-0.8 was used to denote indeterminate genotypic sex.

§ Duplicates / monozygote twins were identified by identity-by-descent (PI HAT) > 0.95.

Filtering for phase 1 JHS may be slightly under-reported as sequencing centers may have filtered samples prior to transfer to the TOPMed Informatics Research Core.

EST = Estonia, FIN = Finland, JHS = Jackson Heart Study

Supplementary Table 2: Variant counts by minor allele frequency across sequenced/imputed and variants across the cohorts (post quality control).

Cohort	Allele Count Bin	Variants (N)	Total variants by dataset
JHS	AC 1	26850162	75,803,412
	AC 2	9065985	
	AC 3 - MAF 0.001	11585033	
	MAF 0.001 - 0.01	13652879	
	MAF 0.01 - 0.05	6580650	
	MAF 0.05 - 0.5	8068703	
EST	AC 1	11692339	31,947,064
	AC 2	3191697	
	AC 3 - MAF 0.001	2740143	
	MAF 0.001 - 0.01	5457897	
	MAF 0.01 - 0.05	2709755	
	MAF 0.05 - 0.5	6155233	
FIN WGS Imputation Panel*	AC 1	0	15,527,751
	AC 2	0	
	AC 3 - MAF 0.001	2502426	
	MAF 0.001 - 0.01	4258578	
	MAF 0.01 - 0.05	2740659	
	MAF 0.05 - 0.5	6026088	
FIN Imputed Array*	AC 1	0	12,256,569
	AC 2	0	
	AC 3 - MAF 0.001	701390	
	MAF 0.001 - 0.01	3410119	
	MAF 0.01 - 0.05	2513638	
	MAF 0.05 - 0.5	5631422	

*Only variants with AC > 2 were included in the FIN WGS imputation panel and FIN imputed array data used in analysis.

Supplementary Table 3: Sample-level summary stats from the data post-quality control.

	Metric‡	EST (WGS)	JHS (WGS)	FIN (WGS)	FIN (Imputation)
**Sample-level Genotype information**	Samples sequenced or genotyped	2,284	3,418	2,690	27,344
	Coverage	28.5 [5.6]	37.6 [4.8]	30.4 [4.1]	NA
**Phenotype Information** *†	Age	47 [18]	63 [13]	49 [13]	48 (13)
	Women	49%	63%	51%	53%
	Lp(a)-C [mg/dL]	7 [5-9]; 2169	7 [5-11]; 2598	NA	NA
	Lp(a) [mg/dL]	NA	46 [24-79]; 2832	4.09 [2.3-11.3]; 591	4.7 [2.4-10.4]; 7064
	LDL [mg/dL]	106 [86-132]; 2169	99 [78-122]; 2358	NA	132 [108-158]; 23387
	HDL [mg/dL]	57 [47-68]; 2169	51 [43-61]; 2937	NA	54 [45-64]; 23797
	TC [mg/dL]	214 [186-248]; 2169	201 [175-229]; 2358	NA	213 [186-242]; 23797
	TG [mg/dL]	118 [86-166]; 2169	91 [67-127]; 2937	NA	105 [76-154]; 23796
	On lipid-lowering drug therapy	239 (11%)	381 (13%)	NA	1526 (6.4%)

* Phenotypic statistics reported for the WGSes reflect individuals present after sample quality control who also had KIV2-CN direcly genotyped

† Lipid values for EST and JHS are from Atherotech; LDL values are from the "True LDL" field, and not the "Total LDL" field in the Atherotech results

‡ Count data are represented as N(%) and continuous data are represented as median[IQR], except age and coverage are expressed as mean[SD]

Supplementary Table 4: Structural variants found overlapping the LPA gene.

Count	Chr	Pos	End	Type	Length	AC JHS	AC EST	AC FIN	Notes*
1	6	161032565	161067901	CNV	5543	ALL	ALL	ALL	KIV2-CN
2	6	160700001	162000000	DUP	1.3Mb	1	0	0	Whole gene
3	6	160987265	161001648	DEL	14,384	2	0	0	KIV8
4	6	161001972	161004287	DEL	2,316	4	1	1	Intronic
5	6	161011567	161015974	DEL	4,408	1	0	0	KIV5 / KIV6
6	6	161023901	161032564	DUP	8,664+*	1	0	0	Intron 17 / KIV2
7	6	161028001	161032564	DEL	4,564+*	37 (MAF 0.5%)	0	0	Intron 17 / KIV2
8	6	161088114	161088883	DEL	770	1	0	0	Upstream
9	6	161088264	161090038	DEL	1,775	2	0	0	Upstream

* Note: only KIV2-CN displays association with Lp(a) phenotypes. Other CNVs are either too rare for power in association, or in the case of CNV #7 (MAF 0.5%), simply do not show association with Lp(a) or Lp(a)-C in JHS carriers.

Supplementary Table 5: KIV2-CN distributions in the WGSes.

	mean(sd)	min-max	mean(sd)*
**JHS**	38.5 (7.4)	16.8-75.7	38.5 (7.4)
**EST**	39.7 (7.0)	12.0-63.3	43.7 (6.2)
**FIN**	45.2 (8.2)	16.1-84.6	
**Overall**	40.1 (8.1)	12.0-84.6	

* p-value of difference between African Americans and Europeans is 0.6

Supplementary Table 6A: Independent, genome-wide significant variants for Lp(a)-C in EST.

Step	Conditioning on:	Top Variant	Conseq.	Gene	P-val			Beta [SD units]			MAF	
					JHS Step 0	EST Step 0	EST Current Step	JHS Step 0	EST Step 0	EST Current Step	JHS	EST
0	-	rs74617384	Intron Variant	LPA	3.00E-12	2.20E-60	2.20E-60	0.92	0.0009	0.0009	0.013	0.05
1	0 + KIV2-CN	rs140570886	Intron Variant	LPA	0.012	5.80E-55	3.30E-32	0.41	1.69	1.28	0.0075	0.017
2	1 + rs140570886	rs74617384	Intron Variant	LPA	3.00E-12	2.30E-60	1.50E-32	0.92	0.0009	0.81	0.013	0.049
3	2 + rs74617384	6:161055991 _C/T	Intron Variant	LPA	NA	6.10E-14	2.70E-13	NA	0.0015	1.48	NA	0.004

Supplementary Table 6B: Independent, genome-wide significant variants for Lp(a)-C in JHS.

Step	Conditioning on:	Top Variant	Conseq.	Gene	P-val			Beta [SD units]			MAF	
					EST Step 0	JHS Step 0	JHS Current Step	EST Step 0	JHS Step 0	JHS Current Step	EST	JHS
0	-	rs138429428	Intron Variant	LPA	NA	5.10E-36	5.10E-36	NA	1.008	1.008	NA	0.032
1	0 + KIV2-CN	rs138429428	Intron Variant	LPA	NA	5.10E-36	7.20E-23	NA	1.008	0.76	NA	0.033
2	1 + rs138429428	rs75143493	Intergenic Variant	-	NA	5.60E-17	3.50E-13	NA	1.037	0.833	NA	0.013

Supplementary Table 7: Interaction associations of top three Lp(a)-C genome-wide significant KIV2-CN modifiers variants. These three top KIV2-CN modifier variants were identified after LD-clumping in plink using the meta-analyzed interaction p-value from JHS and EST ("META_INTERACTION_PVALUE" in table below) and using an r-squared threshold of 0.25.

	**Variant 1**	**Variant 2**	**Variant 3**
**rsID**	rs13192132	rs1810126	rs1740445
**GENE**	LPA	SLC22A3	NA
**Consequence**	intron_variant	3_prime_UTR_variant	intergenic_variant
**META_INTERACTION_PVALUE***	1.73E-15	6.84E-14	6.35E-09
**META_INTERACTION_BETA***	0.0233	-0.0231	0.0158
**META_INTERACTION_SE***	0.0029	0.0031	0.0027
**JHS_KIV2CN_INTER_PVALUE***	6.52E-07	8.14E-05	9.35E-06
**JHS_KIV2CN_INTER_BETA***	0.0221	-0.0199	0.0166
**JHS_KIV2CN_INTER_SE***	0.0044	0.0050	0.0037
**EST_KIV2CN_INTER_PVALUE***	6.26E-10	1.72E-10	1.77E-04
**EST_KIV2CN_INTER_BETA***	0.0242	-0.0250	0.0149
**EST_KIV2CN_INTER_SE***	0.0039	0.0039	0.0040
**NS_META**	5186	5186	5175
**MAF_META_Mean**	0.2385	0.2306	0.3768
**NS_EST**	2249	2249	2238
**NS_JHS**	2937	2937	2937
**MAF_EST**	0.3495	0.3617	0.4151
**MAF_JHS**	0.1536	0.1302	0.3476

* Beta, SE, and P refer to the "KIV2-CN * SNP" term in the following linear model: normalized(Lp(a)-C) ~ KIV2-CN + SNP + KIV2-CN * SNP + cond_SNP + covariates (which includes PC1-5, fasting, age, and sex in both cohorts, and additionally sequencing batch for EST)

Supplementary Table 8: Association of the three top interaction variants with KIV2-CN and normalized Lp(a)-C in EST and JHS.

Beta*†	SE*	P*	Pearson Correlation with KIV2-CN (r^2)	Inter_SNP	Cohort	Outcome
0.696	0.223	1.79E-03	4.83E-03	rs13192132	EST	KIV2-CN
0.082	0.224	0.71	1.20E-05	rs1810126		
0.430	0.219	4.98E-02	2.24E-03	rs1740445		
3.198	0.281	2.94E-29	4.99E-02	rs13192132	JHS	
-0.026	0.302	0.93	3.95E-05	rs1810126		
1.662	0.217	2.52E-14	2.17E-02	rs1740445		
-0.104	0.031	8.59E-04	-	rs13192132	EST	Lp(a)-C
0.212	0.031	1.08E-11	-	rs1810126		
-0.156	0.030	3.17E-07	-	rs1740445		
-0.176	0.040	8.36E-06	-	rs13192132	JHS	
0.213	0.041	2.91E-07	-	rs1810126		
-0.104	0.030	5.51E-04	-	rs1740445		

* Association is from the following linear model: Outcome (KIV2-CN or Lp(a)-C) ~ Inter_SNP + covariates (which includes PC1-5, fasting, age, and sex in both cohorts, and additionally sequencing batch for EST)
† Beta is in units of copy number when outcome is KIV2-CN and SD of Lp(a)-C when outcome is Lp(a)-C

Supplementary Table 9: Sensitivity analysis for top KIV2-CN modifier variants. Association of the three top interaction terms (in the full interaction model) conditioned on genome-wide significant, independent variants for each cohort, in "cond_SNP" column.

Beta [SD Lp(a)-C / KIV2-CN / inter_SNP allele]*	SE*	P*	cond_SNP†	inter_SNP‡	Cohort
0.02210	0.00443	6.52E-07	NA	rs13192132	JHS
0.01813	0.00437	3.43E-05	6:161079599:A:C		
0.01994	0.00442	6.60E-06	6:160946747:T:G		
0.01663	0.00375	9.35E-06	NA	rs1740445	
0.01597	0.00367	1.42E-05	6:161079599:A:C		
0.01597	0.00372	1.82E-05	6:160946747:T:G		
-0.01991	0.00505	8.14E-05	NA	rs1810126	
-0.01053	0.00511	3.93E-02	6:161079599:A:C		
-0.02116	0.00501	2.45E-05	6:160946747:T:G		
0.02417	0.00389	6.26E-10	NA	rs13192132	EST
0.01935	0.00380	3.79E-07	6:161013013:T:C		
0.01407	0.00402	4.76E-04	6:160997118:A:T		
0.02335	0.00390	2.50E-09	6:161055991:C:T		
0.01487	0.00396	1.77E-04	NA	rs1740445	
0.01114	0.00385	3.86E-03	6:161013013:T:C		
0.01209	0.00390	1.95E-03	6:160997118:A:T		
0.01216	0.00401	2.45E-03	6:161055991:C:T		
-0.02495	0.00389	1.72E-10	NA	rs1810126	
-0.01680	0.00390	1.74E-05	6:161013013:T:C		
-0.01613	0.00408	8.10E-05	6:160997118:A:T		
-0.02558	0.00388	5.16E-11	6:161055991:C:T		

* Beta, SE, and P refer to the "KIV2-CN x inter_SNP" term in the following linear model: normalized(Lp(a)-C) ~ KIV2-CN + inter_SNP + KIV2-CN x inter_SNP + cond_SNP + covariates (covariates include PC1-5, fasting, age, and sex in both cohorts, and additionally sequencing batch for EST).
† cond_SNP refers to the SNP conditioned on. If this field is "NA" no SNP was conditioned on.
‡ inter_SNP refers to one of the three top interaction SNPs tested.

Supplementary Table 10: Variance explained by the genetic instruments across Lp(a) and Lp(a)-C phenotypes in the 3 cohorts and multiplicative adjustments used to make each instrument.

Cohort	Phenotype	Genetic Instrument	Variance in phenotype explained by genetic instrument (%)*	Beta of normalized, raw genetic instrument with phenotype used in adjustment, Units: SD Phenotype / SD Genetic Instrument (SE); P-Value†	For KIV2-CN, association of raw KIV2-CN with normalized phenotype (Phenotype ~ raw_KIV2-CN + covariates), Units: SD Phenotype / KIV2-CN (SE); P-Value
FIN	Lp(a)	GRS	30.1	0.543 (0.0102); <1e-300	-
FIN	Lp(a)	KIV2-CN	17.5	-0.404 (0.0108); 2.85e-274	-0.083 (0.0022); 3.15e-283
FIN	Lp(a)	GRS+KIV2-CN	47.0	0.676 (0.008854); <1e-300	-
JHS	Lp(a)	GRS	22.0	0.473 (0.017); 9.3e-150	-
JHS	Lp(a)	KIV2-CN	26.1	-0.512 (0.016); 3.3e-193	-0.069 (0.0022); 1.41e-191
JHS	Lp(a)	GRS+KIV2-CN	48.9	0.711 (0.014); <1e-300	-
JHS	Lp(a)-C	GRS	7.3	0.268 (0.0186); 9.95e-46	-
JHS	Lp(a)-C	KIV2-CN	13.6	-0.370 (0.017); 2.56e-92	-0.049 (0.0024); 1.82e-87
JHS	Lp(a)-C	GRS+KIV2-CN	20.0	0.453 (0.017); 6.22e-136	-
EST	Lp(a)-C	GRS	9.8	0.315 (0.020); 1.13e-52	-
EST	Lp(a)-C	KIV2-CN	11.9	-0.351 (0.020); 1.95e-64	-0.051 (0.0028); 1.81e-67
EST	Lp(a)-C	GRS+KIV2-CN	19.7	0.450 (0.019); 3.71e-110	-

* Variance explained by covariates has been subtracted out of this value

†Beta is from the model: normalized_Phenotype ~ normalized_raw_Genetic_Instrument+covariates, in units of SD Phenotype / SD Genetic Instrument

Supplementary Table 11: Mendelian randomization analysis in Finland across incident clinical phenotypes. Three genetic instruments were used: a weighted genetic risk score using variants conditioned on KIV2-CN at a 4Mb window around LPA (GRS), a KIV2-CN score, and a combined GRS+KIV2-CN score, in addition to the Lp(a) phenotype itself.

HR*	se(Beta)*	Pr(>|z|)*	Incident Phenotype	Instrument*	N cases	N total
**1.30**	**0.048**	**6.46E-08**	**Cardiovascular disease**	**GRS**	**1459**	**21022**
**1.36**	**0.057**	**7.56E-08**	**Coronary heart disease**	**GRS**	**1056**	**21207**
**1.25**	**0.045**	**6.83E-07**	**Coronary heart disease**	**GRS+KIV2-CN**	**1056**	**21207**
**0.79**	**0.048**	**7.51E-07**	**Diabetes**	**Lp(a)**	**444**	**6078**
**1.18**	**0.039**	**1.41E-05**	**Cardiovascular disease**	**GRS+KIV2-CN**	**1459**	**21022**
**1.24**	**0.051**	**1.82E-05**	**Acute coronary syndrome**	**GRS+KIV2-CN**	**826**	**21320**
**1.30**	**0.064**	**3.61E-05**	**Acute coronary syndrome**	**GRS**	**826**	**21320**
**1.30**	**0.076**	**6.76E-04**	**Myocardial infarction**	**GRS**	**580**	**21377**
**0.85**	**0.050**	**8.05E-04**	**Heart failure**	**Lp(a)**	**411**	**6322**
**1.23**	**0.061**	**8.19E-04**	**Myocardial infarction**	**GRS+KIV2-CN**	**580**	**21377**
1.27	0.075	1.35E-03	Stroke	GRS	598	21424
1.16	0.050	3.71E-03	Coronary heart disease	Lp(a)	426	6317
1.10	0.042	0.03	Cardiovascular disease	Lp(a)	590	6271
1.20	0.086	0.04	Acute coronary syndrome	KIV2-CN	826	21320
1.11	0.056	0.06	Acute coronary syndrome	Lp(a)	337	6350
1.12	0.060	0.06	Stroke	GRS+KIV2-CN	598	21424
0.93	0.042	0.07	Cancers	GRS+KIV2-CN	1228	21104
1.13	0.076	0.11	Coronary heart disease	KIV2-CN	1056	21207
0.90	0.071	0.13	Diabetes	KIV2-CN	1212	20313
1.16	0.102	0.15	Myocardial infarction	KIV2-CN	580	21377
0.93	0.053	0.16	Cancers	GRS	1228	21104
1.10	0.067	0.17	Myocardial infarction	Lp(a)	239	6366
0.78	0.183	0.19	Chronic kidney failure	Lp(a)	29	6434
0.91	0.070	0.20	Cancers	KIV2-CN	1228	21104
0.90	0.100	0.31	Stroke	KIV2-CN	598	21424
0.96	0.043	0.35	Diabetes	GRS+KIV2-CN	1212	20313
0.94	0.077	0.44	Heart failure	KIV2-CN	1004	21249
1.06	0.073	0.44	Dementia	GRS+KIV2-CN	409	21622
0.87	0.183	0.46	Chronic kidney failure	GRS+KIV2-CN	64	21602
0.86	0.230	0.52	Chronic kidney failure	GRS	64	21602
1.08	0.121	0.53	Dementia	KIV2-CN	409	21622
1.05	0.091	0.57	Dementia	GRS	409	21622
1.04	0.075	0.58	Dementia	Lp(a)	184	6437
1.03	0.064	0.60	Cardiovascular disease	KIV2-CN	1459	21022
0.98	0.047	0.65	Heart failure	GRS+KIV2-CN	1004	21249
1.02	0.064	0.71	Stroke	Lp(a)	258	6386
1.01	0.058	0.81	Heart failure	GRS	1004	21249
1.01	0.053	0.84	Diabetes	GRS	1212	20313
0.99	0.045	0.86	Cancers	Lp(a)	491	6300
0.97	0.302	0.93	Chronic kidney failure	KIV2-CN	64	21602

*Each genetic instrument has been normalized such that 1 unit increase in the genetic instrument relates to 1 SD increase in Lp(a) phenotype. Association statistics for the Lp(a) phenotypic instrument are also provided in inverse-rank normalized units (SD of Lp(a)).

Supplementary Table 12: Mendelian Randomization using sub-clinical markers of atherosclerosis: Abdominal Aortic Calcium (AAC) and Coronary Artery Calcium (CAC) in Europeans and African Americans. Mendelian randomization was performed using three genetic instruments: a weighted genetic risk score using variants conditioned on KIV2-CN at a 4Mb window around LPA (GRS), a KIV2-CN score, and a combined GRS+KIV2-CN score, and compared to the observational effects (from Lp(a) and/or Lp(a)-C). The genetic instruments were all normalized such that 1 unit increase in the instrument is equal to 1SD increase in Lp(a) or Lp(a)-C (as specified in the "Pheno" column).

Outcome	Pheno	Instrument	Ethnicity	N (total)	Cohorts	Beta	SE	P
CAC	Lp(a)	KIV2-CN	European	2778	FHS;MESA	-0.015	0.033	0.66
			African American	2470	MESA;JHS	0.023	0.031	0.45
		GRS	European	3132	FHS;MESA	0.056	0.025	0.03
			**African American**	**2574**	**MESA;JHS**	**0.097**	**0.037**	**9.20E-03**
		GRS+KIV2-CN	European	2771	FHS;MESA	0.039	0.021	0.06
			African American	2411	MESA;JHS	0.052	0.024	0.03
		Lp(a)	European	2493	MESA	0.021	0.015	0.14
			**African American**	**3221**	**MESA;JHS**	**0.053**	**0.014**	**1.90E-04**
	Lp(a)-C	KIV2-CN	European	3361	FHS;MESA;Amish	0.009	0.035	0.81
			African American	2470	MESA;JHS	0.032	0.043	0.45
		GRS	European	3727	FHS;MESA;Amish	0.055	0.039	0.16
			**African American**	**2574**	**MESA;JHS**	**0.161**	**0.060**	**7.54E-03**
		GRS+KIV2-CN	European	3353	FHS;MESA;Amish	-0.012	0.028	0.68
			African American	2411	MESA;JHS	0.074	0.036	0.04
		Lp(a)-C	European	1508	FHS;Amish	0.004	0.019	0.85
			**African American**	**1701**	**JHS**	**0.067**	**0.021**	**1.30E-03**
AAC	Lp(a)	KIV2-CN	European	1490	FHS	0.000	0.041	0.99
			African American	1700	JHS	0.092	0.039	0.02
		GRS	European	1536	FHS	0.056	0.031	0.07
			African American	1641	JHS	0.098	0.043	0.02
		GRS+KIV2-CN	European	1483	FHS	0.021	0.025	0.39
			**African American**	**1641**	**JHS**	**0.097**	**0.029**	**7.38E-04**
		Lp(a)	**African American**	**1641**	**JHS**	**0.094**	**0.020**	**3.76E-06**
	Lp(a)-C	KIV2-CN	European	1805	FHS;Amish	0.010	0.041	0.81
			African American	1700	JHS	0.128	0.054	0.02
		GRS	European	1858	FHS;Amish	0.088	0.047	0.06
			African American	1641	JHS	0.101	0.077	0.19
		GRS+KIV2-CN	European	1798	FHS;Amish	0.018	0.033	0.58
			**African American**	**1641**	**JHS**	**0.123**	**0.045**	**6.30E-03**
		Lp(a)-C	European	1288	FHS;Amish	0.022	0.018	0.23
			African American	1700	JHS	0.043	0.020	0.03

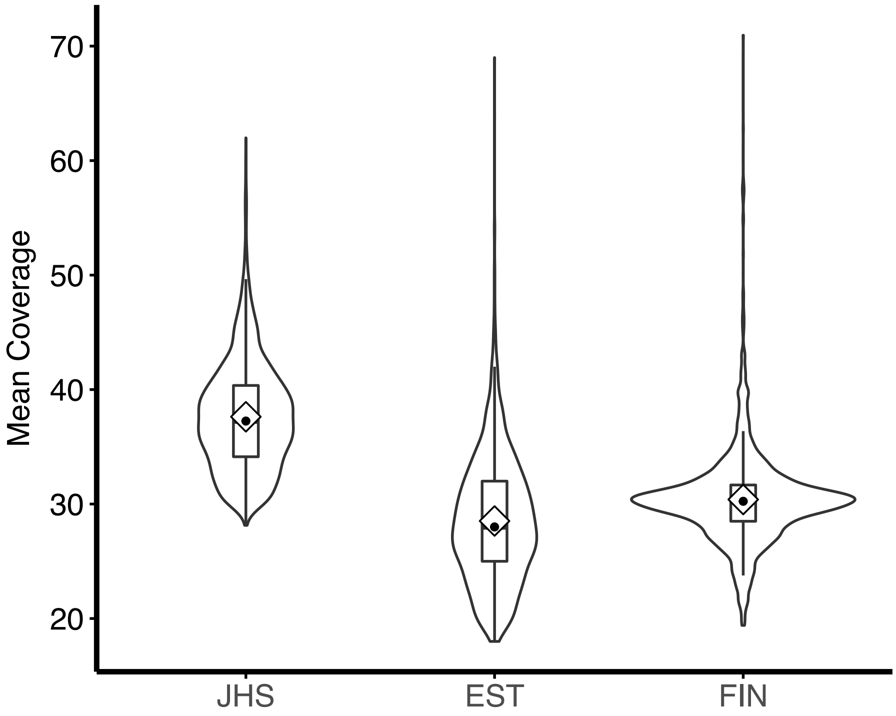

Supplementary Fig. 1 Mean fold coverage in whole genome sequences by cohort.

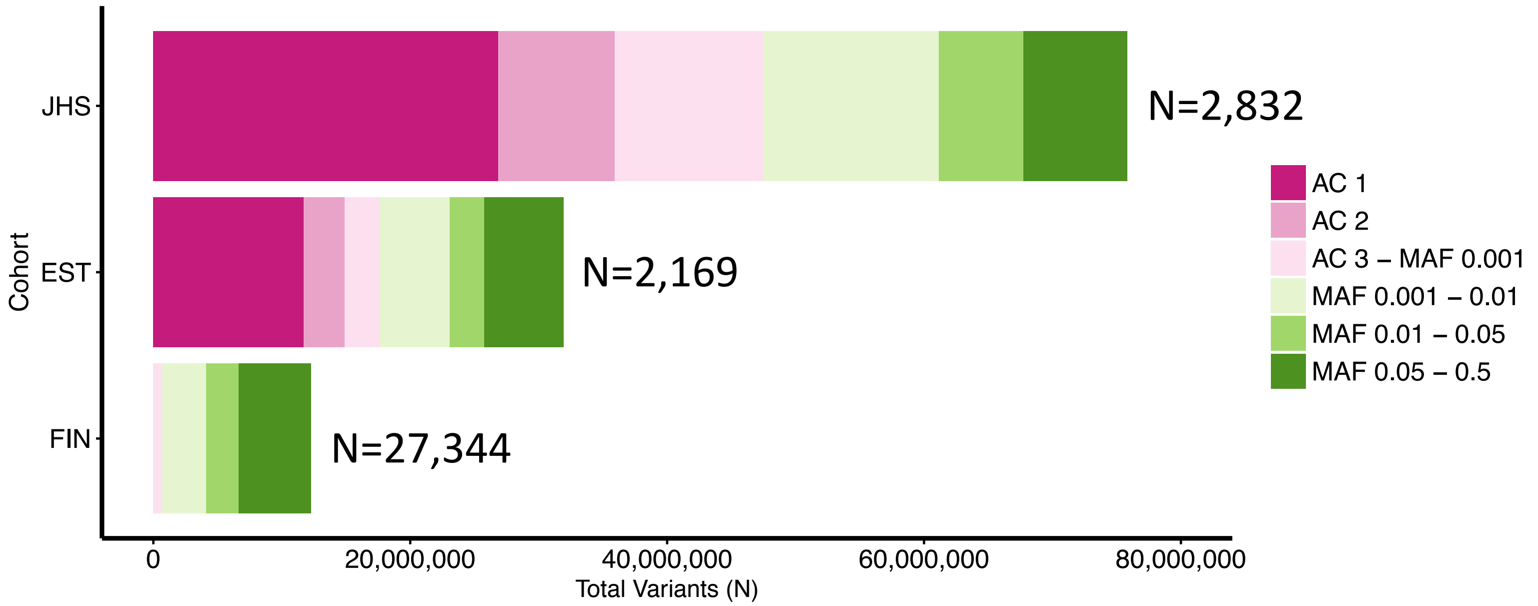

Supplementary Fig. 2 Variant counts by cohort and minor allele frequency (MAF). The total number of variants whole-genome sequenced (JHS, EST) and in the FIN imputation dataset is provided by allele frequency bin. The number of samples in each Cohort are denoted to the right of the bars.

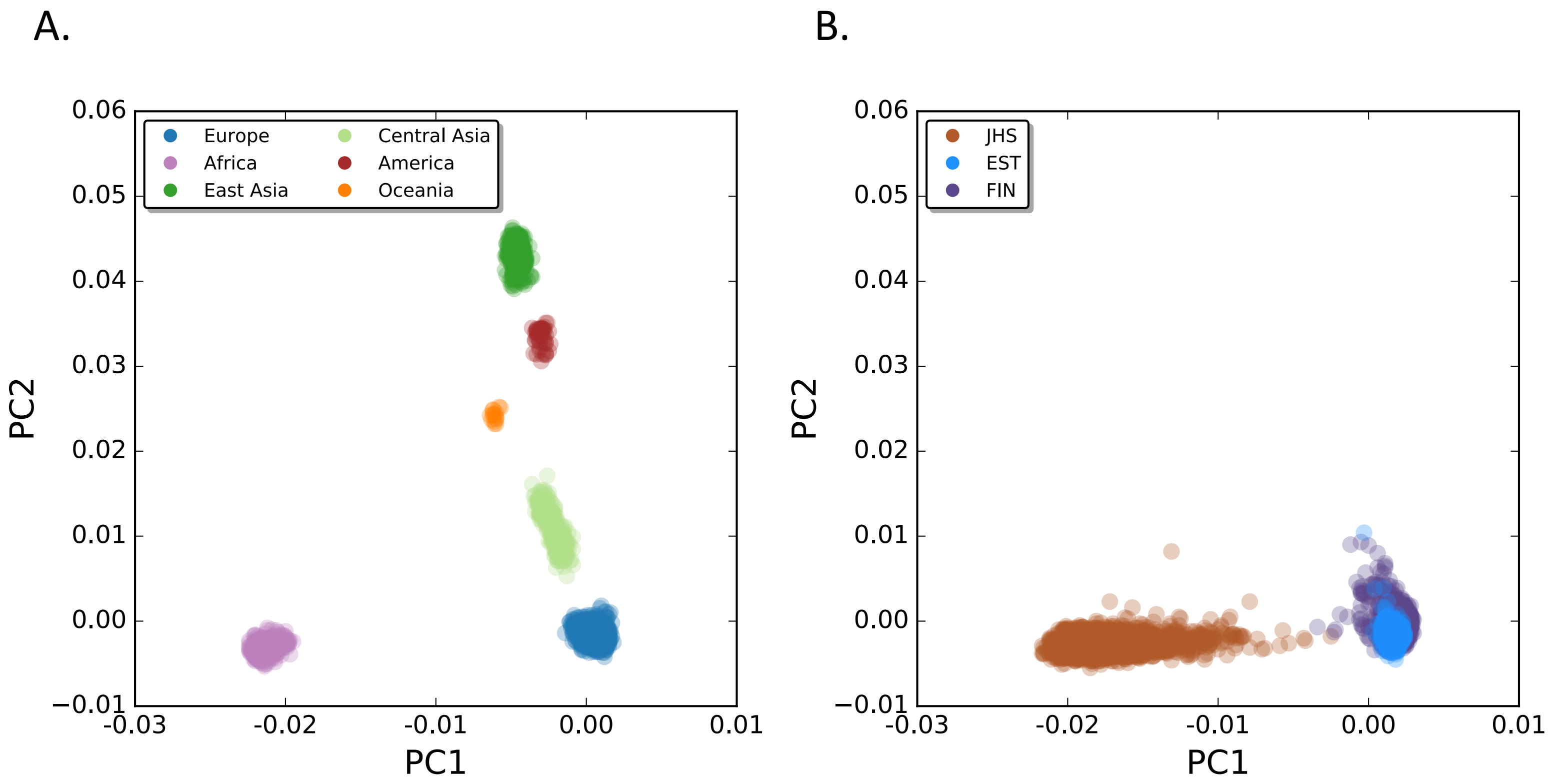

Supplementary Fig. 3 Principle component analyses. Principle component analysis was performed to estimate genetic ancestry by first extracting principle components across unrelated individuals and then using these to estimate principal components of close relatives in the dataset. These principal components were compared to reference samples shown in panel **(A)**, verifying the African American and European clusters of ancestry in the three cohorts **(B)**.

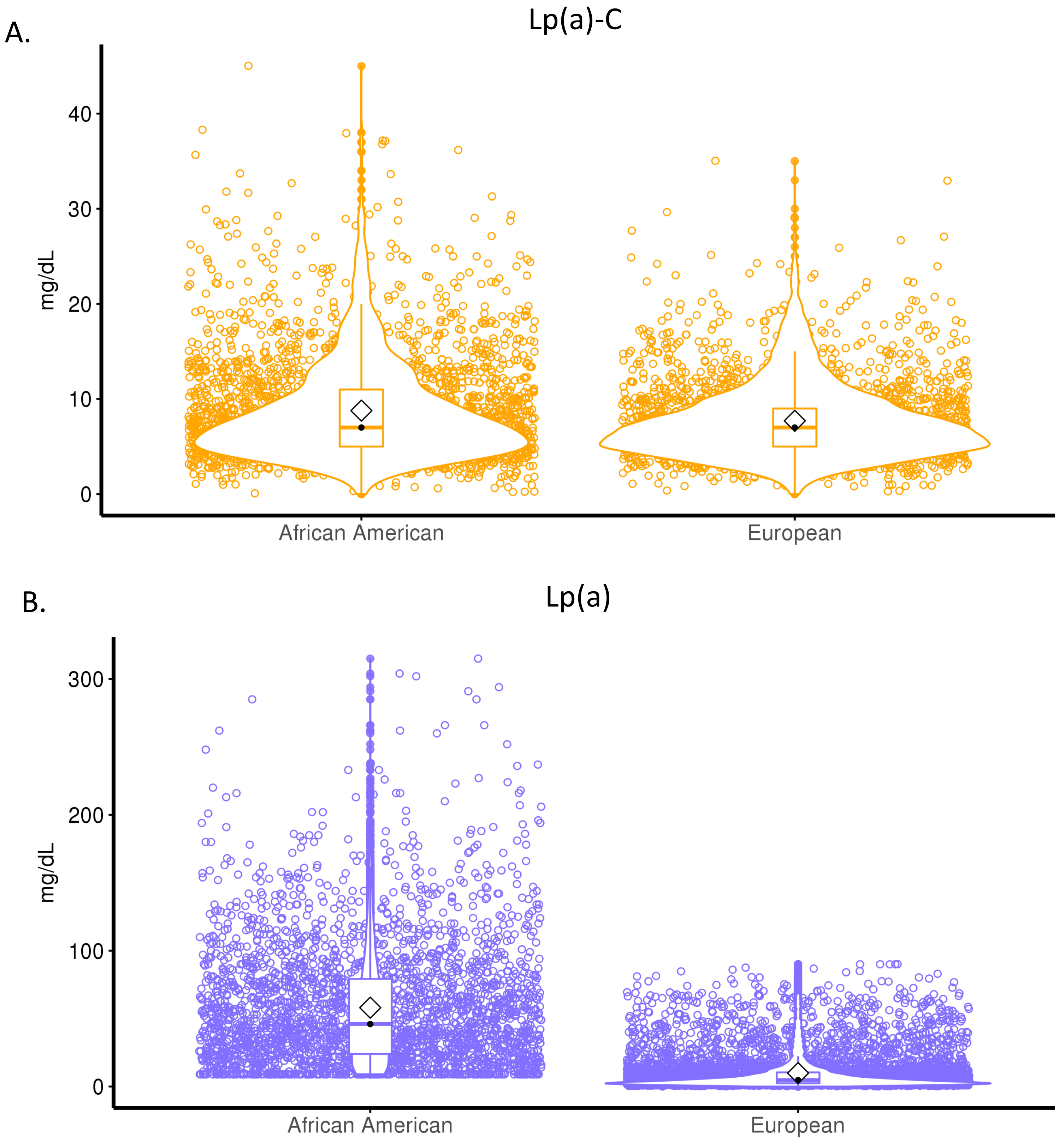

Supplementary Fig. 4 Phenotype distributions (in mg/dL) by ethnicity for **A)** Lp(a)-C and **B)** Lp(a).

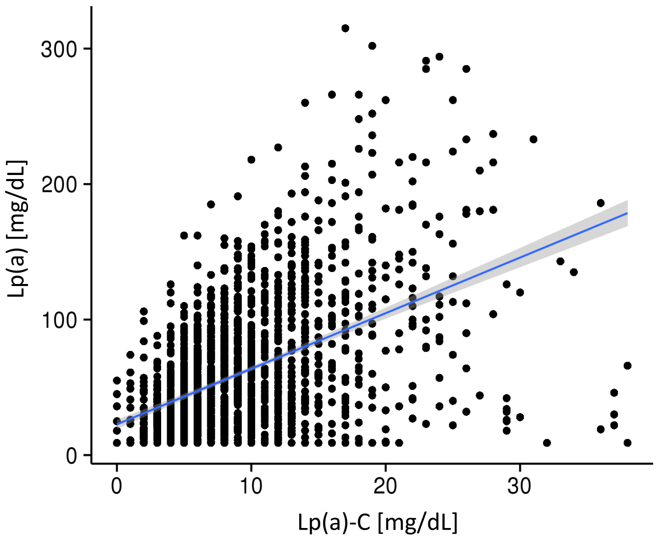

Supplementary Fig. 5 Observational correlation between Lp(a) and Lp(a)-C in 2,832 African American individuals from JHS. Lp(a) and Lp(a)-C are correlated with a Spearman correlation of 0.46 (Beta: 0.44 SD Lp(a)/SD Lp(a)-C, p-value = 2.4e-143).

A.

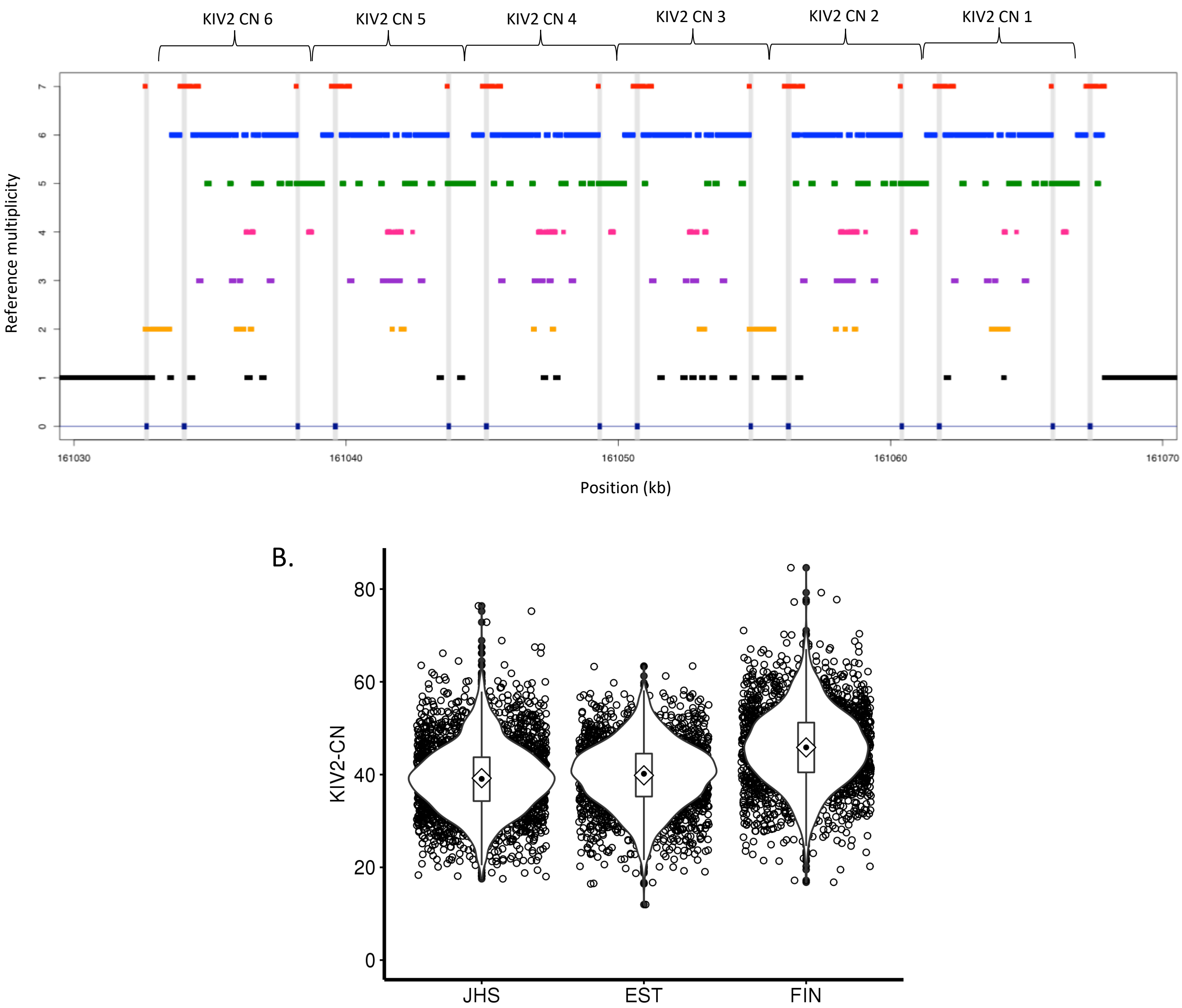

Supplementary Fig. 6 KIV2-CN estimation from whole genome sequences. **A)** The six KIV2-CN repeats in the hg19 reference genome are shown, with the y-axis representing the number of times, ie: multiplicity, with which each k-mer (100b) is present on the reference genome. The blue track at the bottom represents the location on the *LPA* gene, with exons bolded in dark blue and highlighted with gray vertical lines. The regions encoding KIV2 domains are annotated at the top, with each KIV2 domain containing 2 exons and six KIV2 domains being part of the hg19 reference genome. Note: the KIV2 domains excludes the first highlighted exon on the right (part of the KIV1 domain) and the last highlighted exon on the left (part of the KIV3 domain), despite the homology of both of these exons to exons that encode the KIV2 domains (which also results in 7 k-mers noted in the y-axis as opposed to 6). Note: LPA is translated from right to left. **B)** The distribution of directly genotyped KIV2-CN (representing total KIV2-CN across both chromosomes) is shown for each whole-genome sequenced cohort.

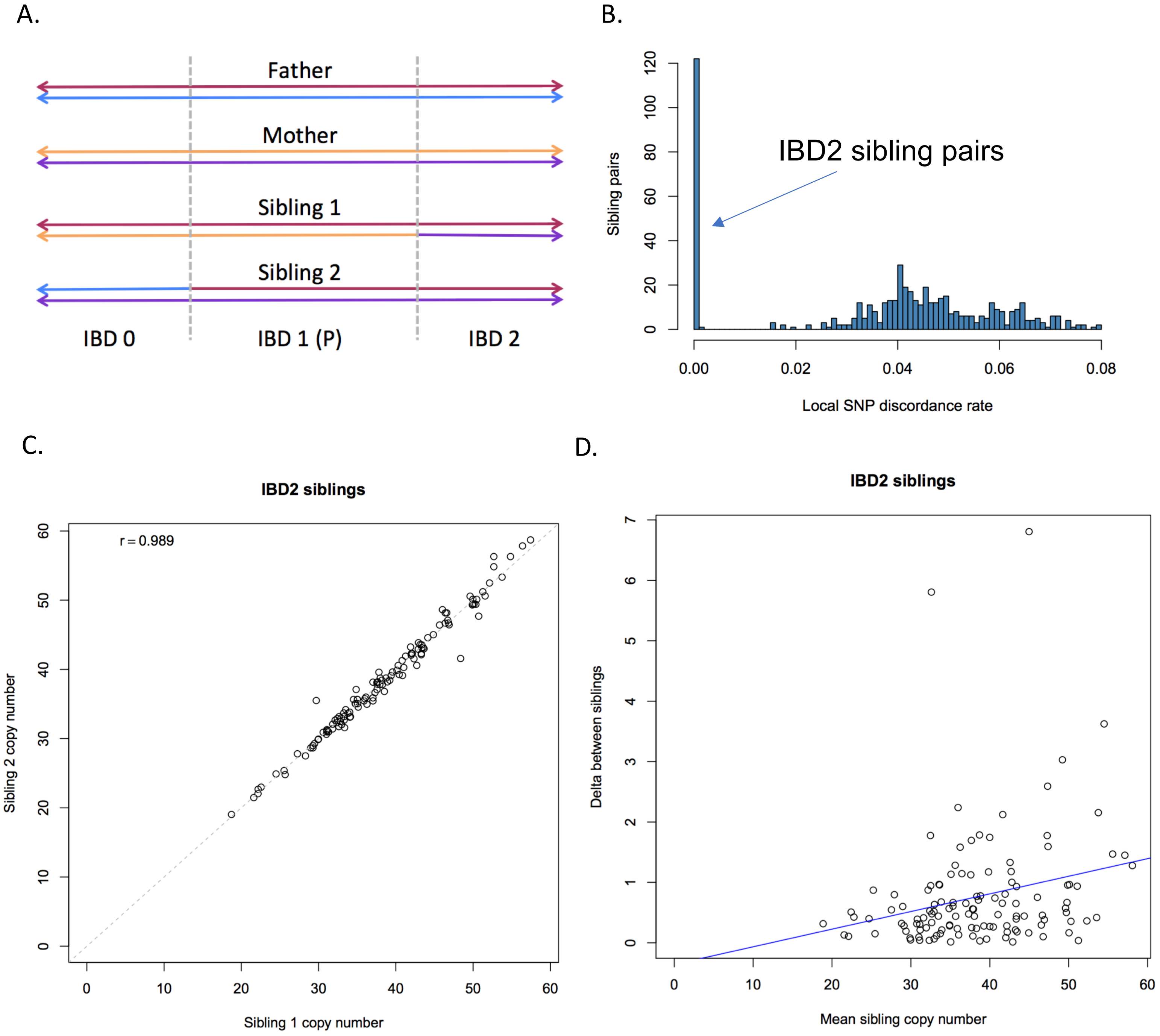

Supplementary Fig. 7 Determining the precision of directly genotyped KIV2-CN using IBD2 siblings in JHS. **A)** To determine the precision of our directly genotyped KIV2-CN, we used sibling pairs in JHS which are 99% identical by descent in both chromosomes (IBD 2) at a 1MB region around the LPA transcription start site, such that both siblings have inherited the same chromosome from their parents at this region. **B)** 123 IBD 2 sibling pairs were identified. Among these sibling pairs, **C)** the spearman correlation coefficient between directly genotyped KIV2-CNs was 0.989, and **D)** the difference in genotyped KIV2-CN between IBD 2 sibling pairs (i.e.: abs(Sibling 1 copy number - Sibling 2 copy number)) was seen to on average increase with mean sibling KIV2-CN.

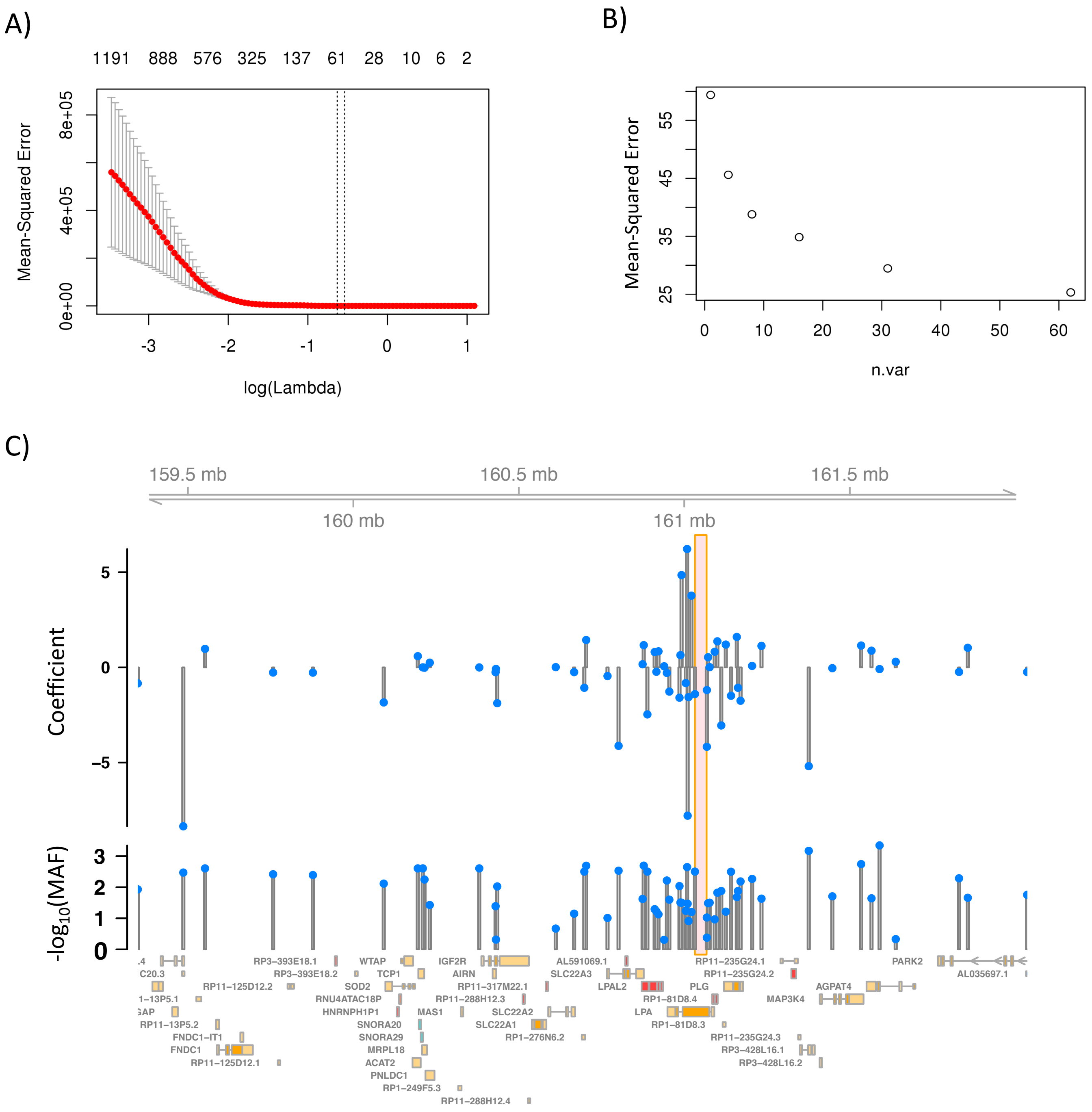

Supplementary Fig. 8 KIV2-CN imputation model. The least absolute shrinkage and selection operator (LASSO) machine learning model was used against an initial set of 7,484 LD-pruned high-quality imputed Finnish variants (imputation quality > 0.8) to predict directly-genotyped KIV2-CN in 1477 WGSed individuals used in the training dataset with 10-fold cross validation. **A)** The mean-squared error and 95% confidence intervals from the LASSO model are plotted against log(Lambda) on the lower x-axis, where Lambda refers to the degree of shrinkage, and the number of variants used in the model are specified on the top of the plot. The vertical dotted lines display the location of the minimum mean-squared error, which occurs with 61 variants in the model. **B)** Using these 61 variants in a random forest model, the mean-squared error is plotted against the number of variants in the model showing exponential decay of mean-squared error as the number of variants approaches 61. **C)** The LASSO coefficients of the 61 variants and their MAF (visualized as –log10(MAF)) are displayed. The orange highlighted region denotes where the KIV2-CN is located.

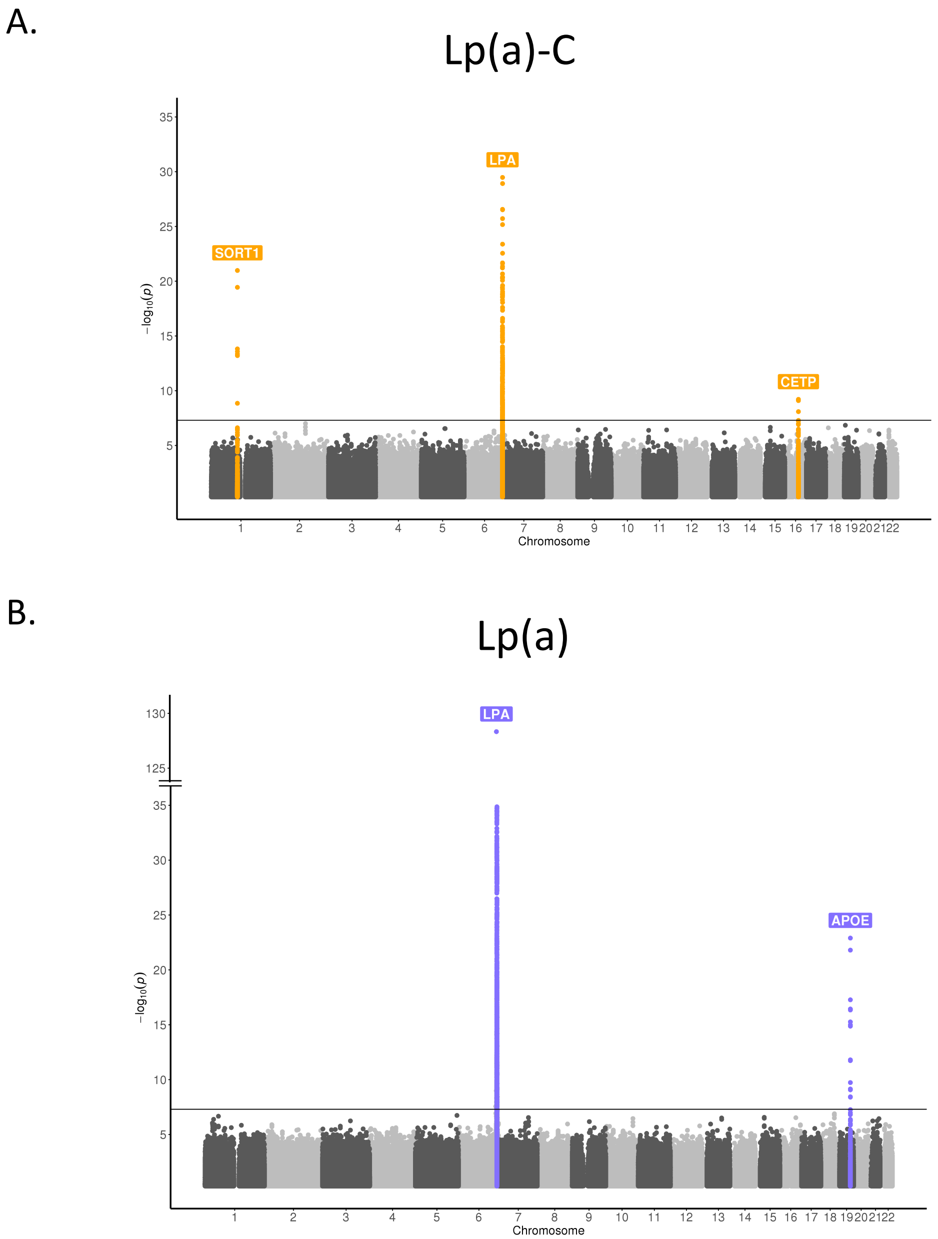

Supplementary Fig. 9 Manhattan plots of meta-analyzed single variant associations with **A)** Lp(a)-C and **B)** Lp(a).

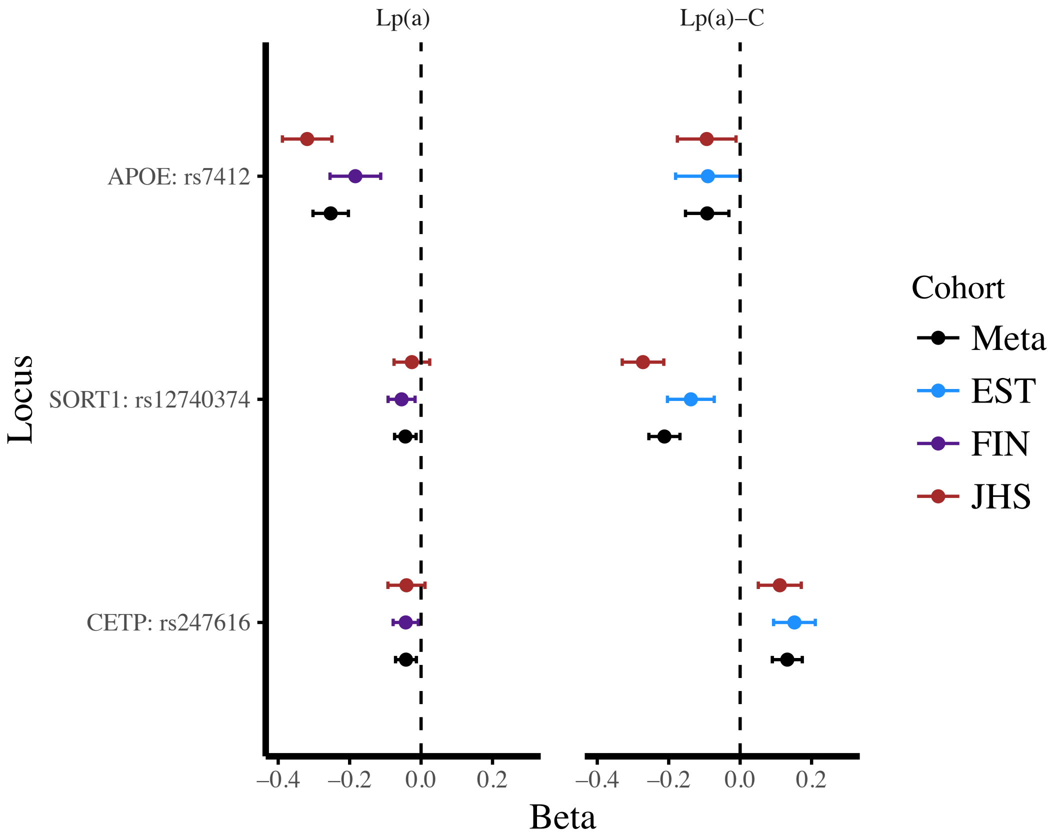

Supplementary Fig. 10 Associations of top 3 non-LPA loci with both Lp(a) and Lp(a)-C, conditioned on KIV2-CN. Betas and 95% confidence intervals for rs7412 at the APOE locus (genome-wide significant for Lp(a)), rs12740374 at the SORT1 locus (genome-wide significant for Lp(a)-C), and rs247616 at the CETP locus (genome-wide significant for Lp(a)-C) are shown by cohort and meta-analyzed.

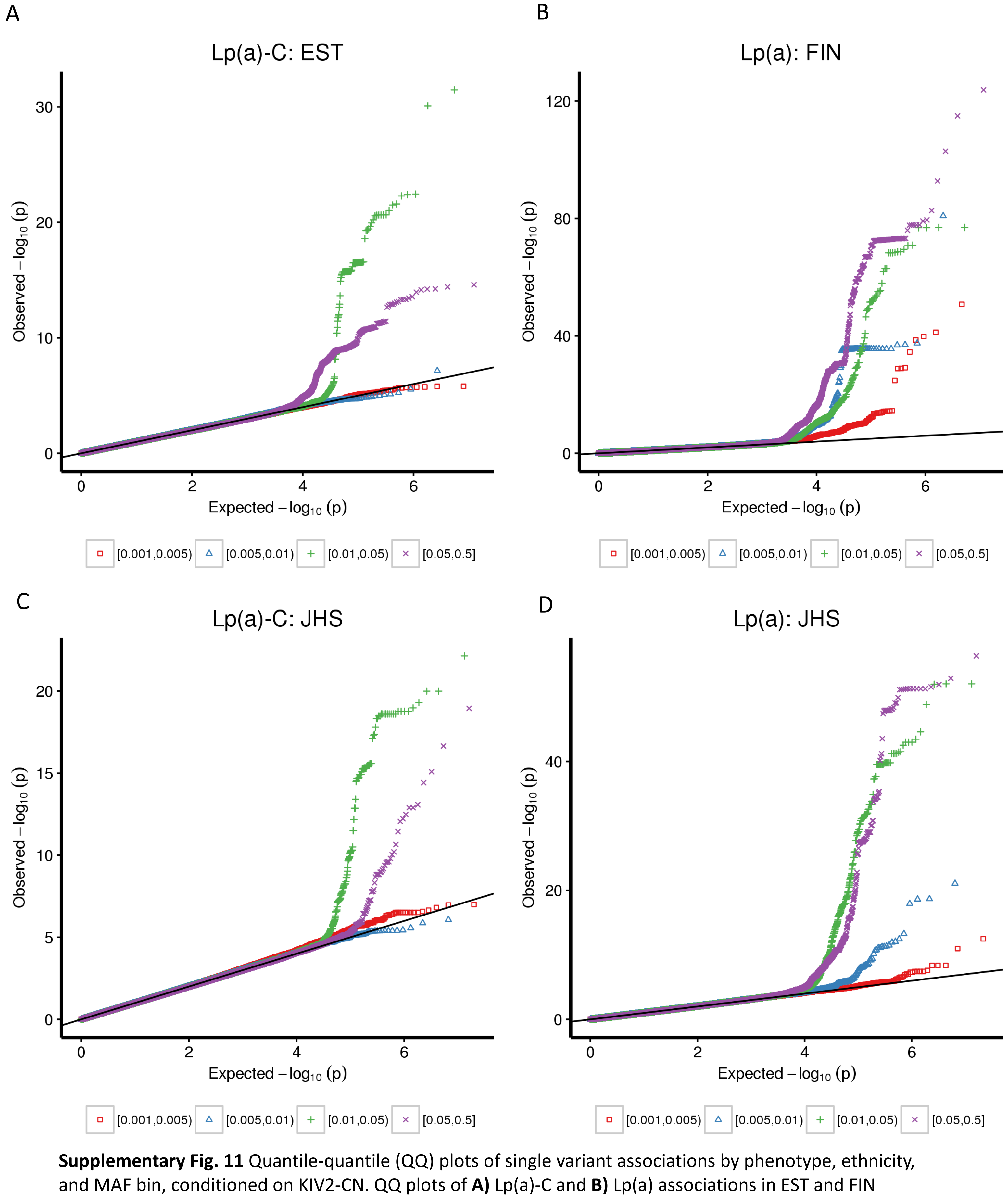

Supplementary Fig. 11 Quantile-quantile (QQ) plots of single variant associations by phenotype, ethnicity, and MAF bin, conditioned on KIV2-CN. QQ plots of **A)** Lp(a)-C and **B)** Lp(a) associations in EST and FIN European cohorts and **C)** Lp(a)-C and **D)** Lp(a) associations in JHS African American cohort are presented by minor allele frequency bin.

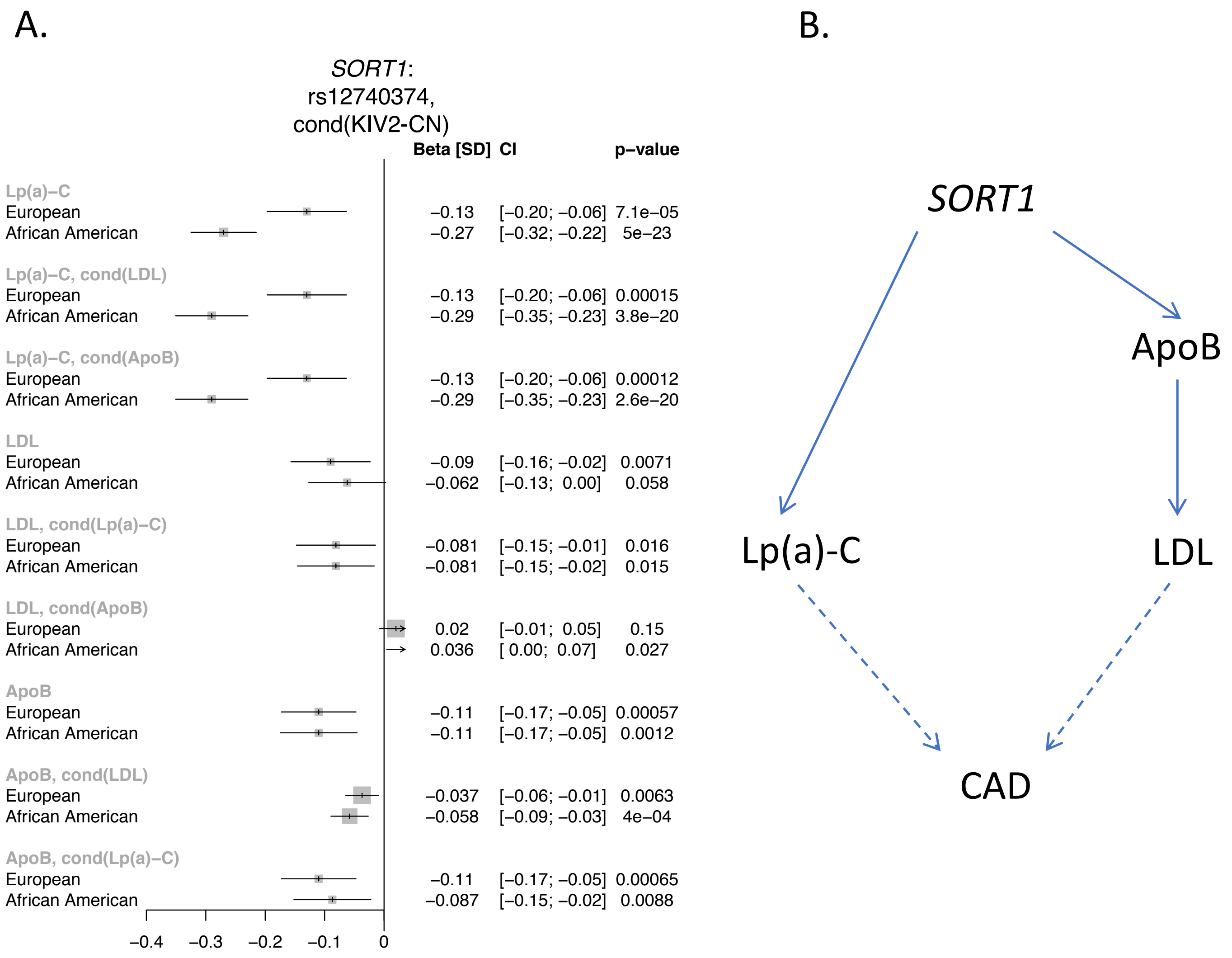

Supplementary Fig. 12 Conditional analysis using rs12740374 at the SORT1 locus shows evidence of independence from ApoB and LDL. **A)** Betas and 95% confidence intervals (in SD of the phenotype used, either Lp(a)-C, LDL, or ApoB), of the top variant at the SORT1 locus, rs12740374, between European and African Americans with Lp(a)-C, LDL, and ApoB conditioned on each other (represented by "cond(Lp(a)-C)", "cond(LDL)", and "cond(ApoB)" shows evidence that **B)** this locus's effect on Lp(a)-C is independent of its effect on ApoB and LDL, both pathways of which are known to influence coronary artery disease (CAD) risk.

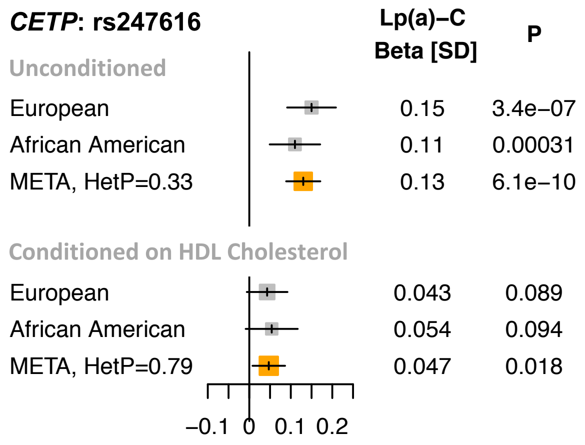

Supplementary Fig. 13 Conditioning the *CETP* locus on HDL cholesterol mitigates its association with Lp(a)-C. Common variants at *CETP* are known to be associated with HDL cholesterol. Here we performed conditional analysis on the lead *CETP* locus variant for Lp(a)-C, rs247616, finding that it is no longer genome-wide significant after conditioning on HDL cholesterol.

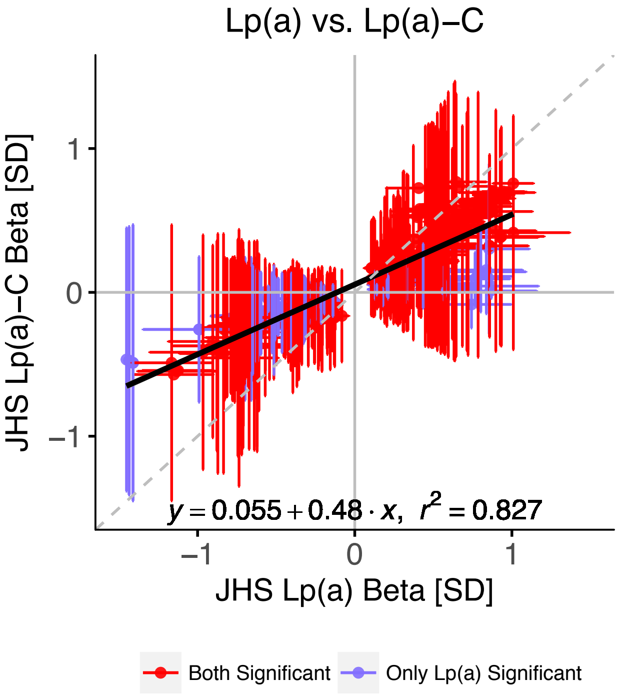

Supplementary Fig. 14 Comparison of Lp(a) and Lp(a)-C phenotypic correlation and genetic correlation from betas in single variant association at the LPA locus in JHS. Showing betas and 95% confidence intervals of 770 variants at a 1MB window around the *LPA* locus which are genome-wide significant (p-value < 5e-8) for either Lp(a)-C or Lp(a), colored by whether there's significance (p-value < 0.05) in both phenotypes (red) or significance with only Lp(a) (purple). Note: all variants that are genome-wide significant with Lp(a)-C show also show evidence of significance (p-value < 0.05) with Lp(a). The four quadrants are separated by solid gray lines and a dotted gray line at y=x is displayed for comparison with the solid black best-fit line. Of note, no variants display opposite effects with the Lp(a) versus Lp(a)-C phenotypes (i.e.: no variant betas in the top left or bottom right quadrants).

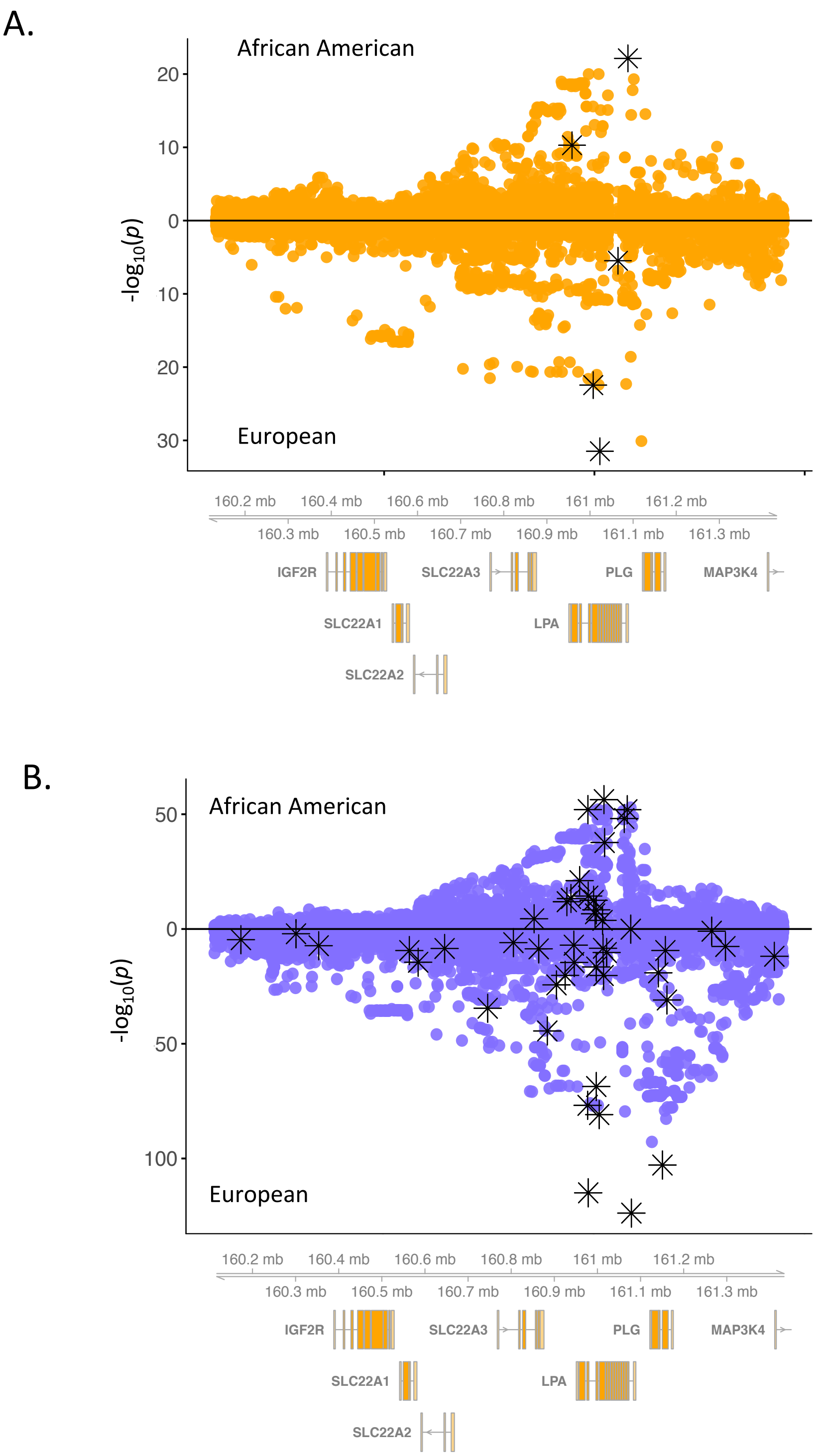

Supplementary Fig. 15 Independent variants associated with Lp(a)-C and Lp(a) at the LPA locus by ethnicity. **A)** Lp(a)-C locus-zoom plots (conditioned on KIV2-CN) for African Americans (top) and Europeans (bottom) are shown, with conditionally independent, genome-wide significant variants displayed with asterisks. **B)** Lp(a) locus-zoom plots (conditioned on KIV2-CN) for African Americans (top) and Europeans (bottom) are shown, with conditionally independent, genome-wide significant variants displayed with asterisks.

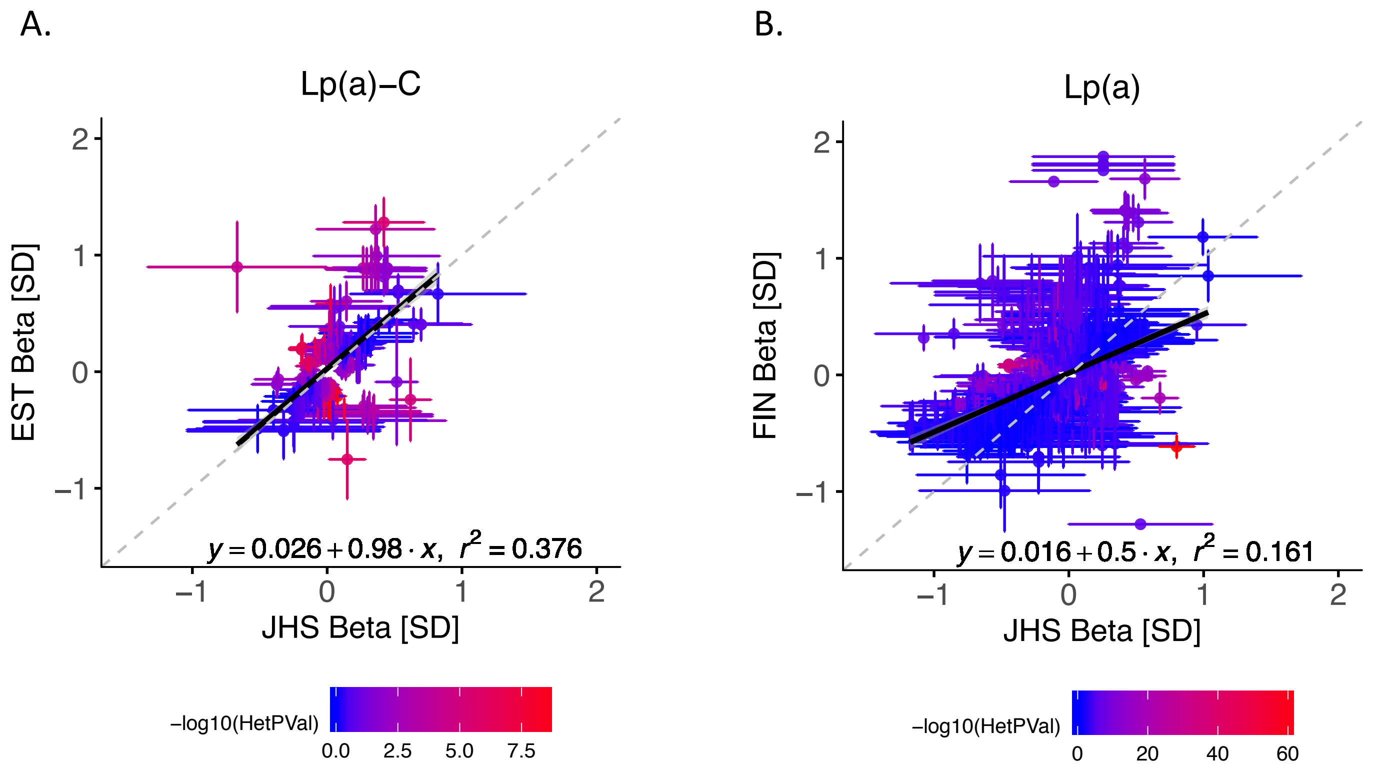

Supplementary Fig. 16 Heterogeneity between ethnicities for sub-threshold significant variants. Within a1MB window around the LPA TSS, we compared betas of association and heterogeneity between African Americans and Europeans separately by phenotype for **A)** Lp(a)-C and **B)** Lp(a), focusing on variants that were sub-threshold significant (p-value < 1e-4) in either ethnicity. Here, each variant is represented as a separate point, with betas (in SD) and 95% confidence intervals for both ethnicities represented. The gray dotted line in both plots represents the unity line, y=x, and serves as a frame of reference to compare the best-fit black line with. Each variant is colored with its heterogeneity p-value (HetPVal), as represented below the plot. For Lp(a)-C, the correlation between betas across ethnicities is more than twice than that for Lp(a) (R2 = 0.376 for Lp(a)-C, R2 = 0.161 for Lp(a)). Furthermore, the best-fit line using this subset of variants is closer to unity for Lp(a)-C (slope of 0.98) than it is for Lp(a) (slope of 0.5), with stronger heterogeneity p-values between ethnicities in Lp(a).

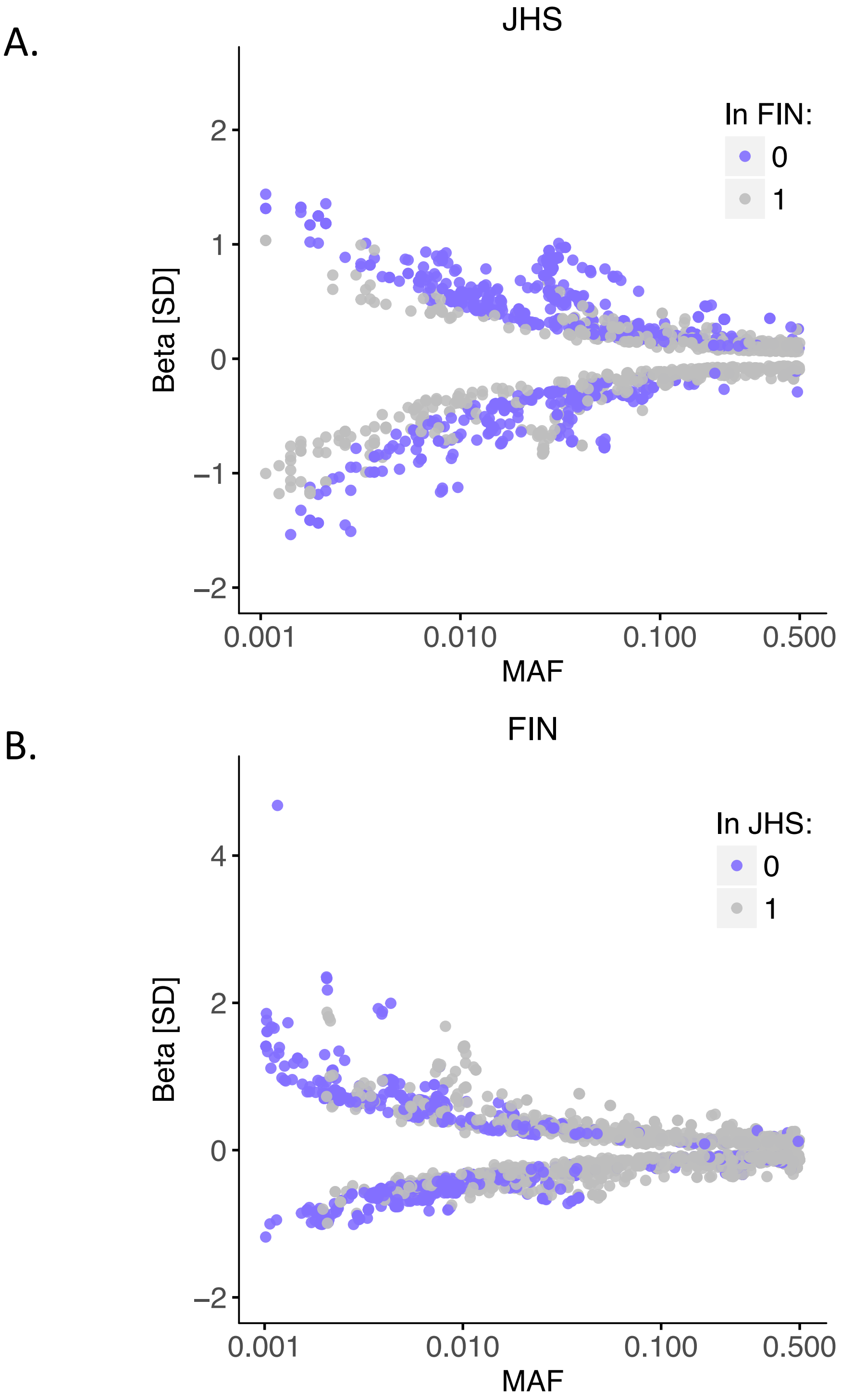

Supplementary Fig. 17 Allele frequency spectrum and Lp(a) effect size of variants at the LPA locus by ethnicity. To further understand the heterogeneity of associations between ethnicities for Lp(a), we looked into the relationship between MAF and Beta within variants at the LPA locus showing some significance (p-value < 1e-2) in **A)** JHS and **B)** FIN. In purple are ethnic-specific variants which are not present with allele frequency > 0.001 in the other analyzed cohort.

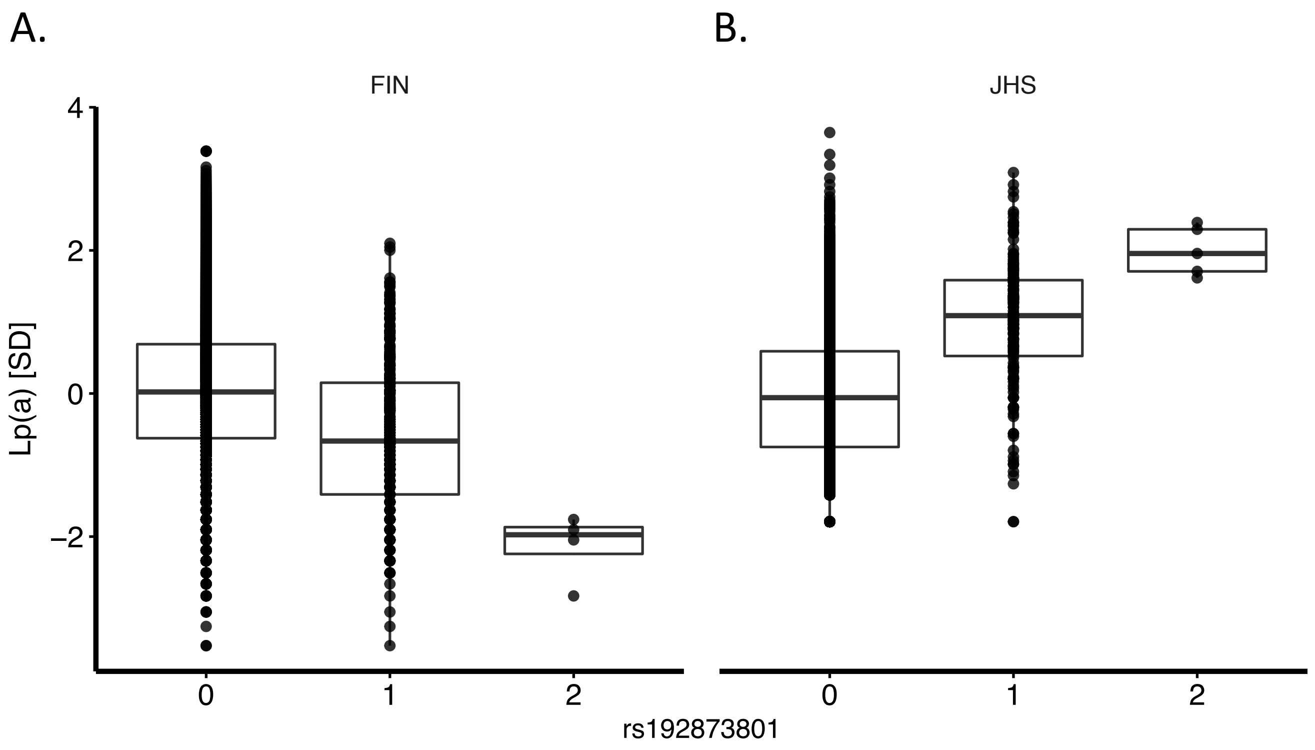

Supplementary Fig. 18 Highlighting rs192873801, a variant with significant heterogeneity across ethnicities. Shown are boxplots by cohort for **A)** FIN and **B)** JHS showing the association of 0, 1, and 2 alternate alleles of rs192873801 with Lp(a) [SD]. rs192873801 is an LPAL2 intronic variant and shows the strongest heterogeneity p-value (HetP= 9.75e-64) for Lp(a) between African American and European ethnicities, resulting in opposite, but genome-wide significant effects in each ethnicity (p-value 2.0e-35, beta -0.61 SD, MAF 0.027 in FIN and p-value 3.8e-32, beta 0.80 SD, MAF 0.028 in JHS).

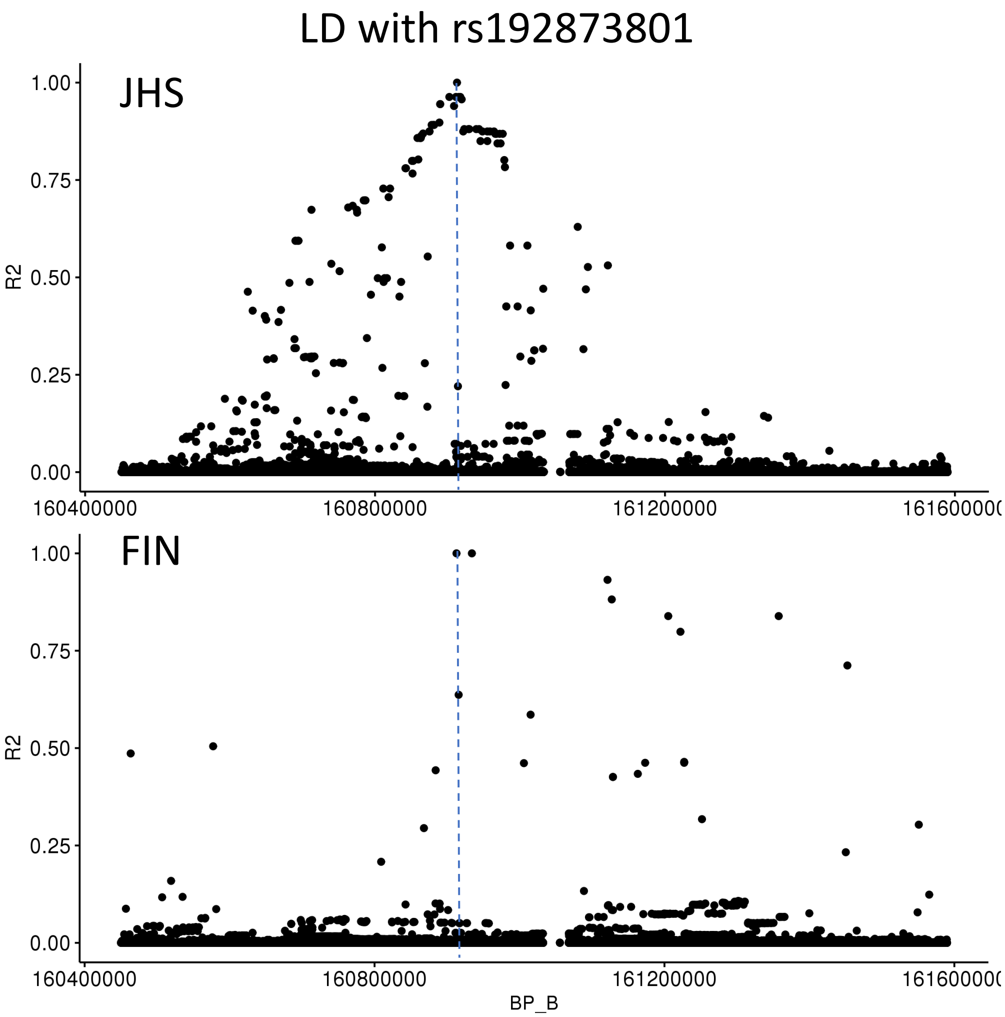

Supplementary Fig. 19 Haplotypes associated with rs192873801 in JHS and FIN. The linkage-disequilibrium R^2 values between rs192873801 and variants nearby are displayed, showing that this variant is on two separate haplotypes in the JHS African Americans versus FIN Europeans.

A.

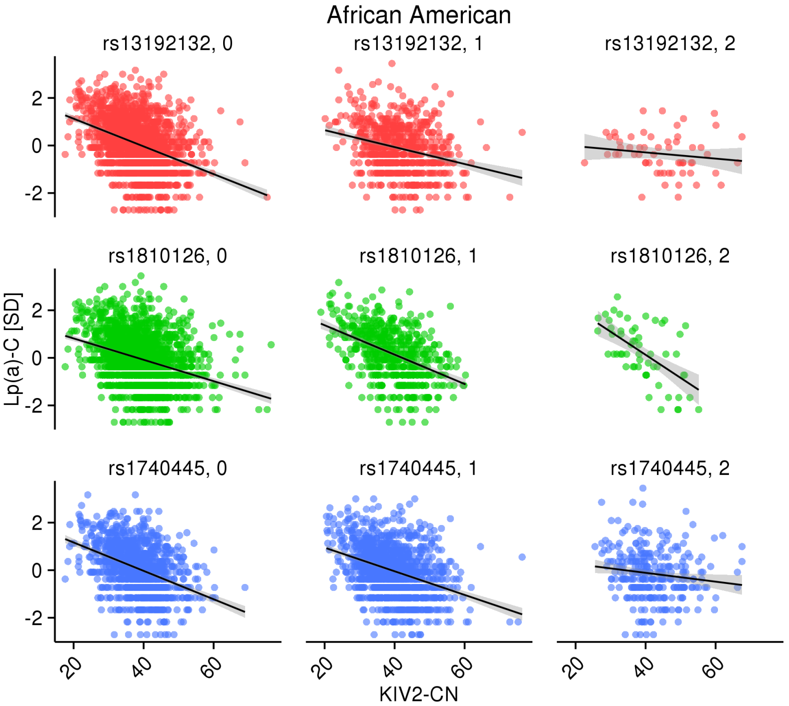

B.

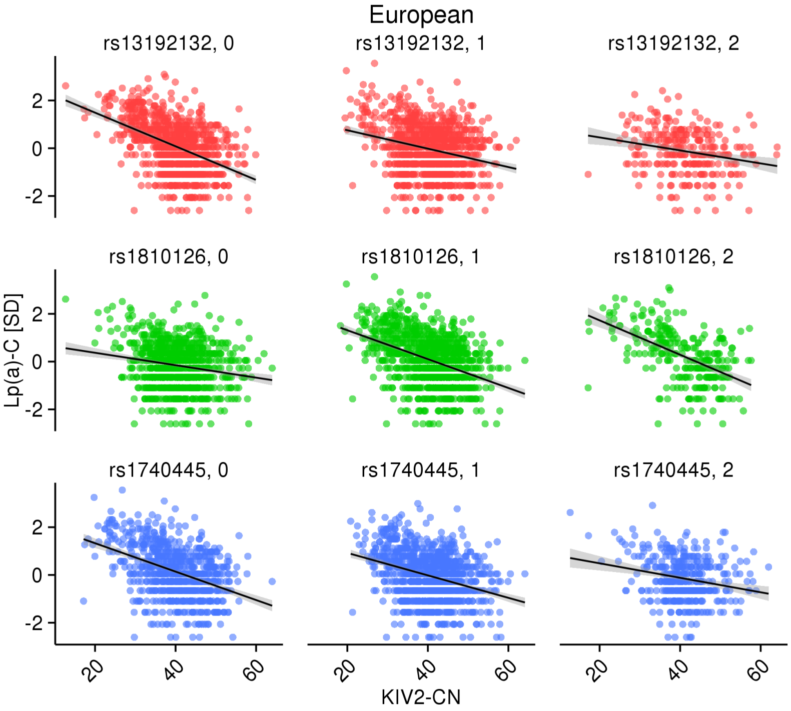

Supplementary Fig. 20 Effects of top 3 independent modifier variants on interaction between Lp(a)-C and KIV2-CN by ethnicity. Shown are the relationships between Lp(a)-C concentrations [SD] and KIV2-CN in carriers of 0, 1, and 2 alleles of rs13192132, rs1810126, and rs1740445 within whole-genome sequenced **A)** African Americans from JHS, and **B)** Europeans from EST.

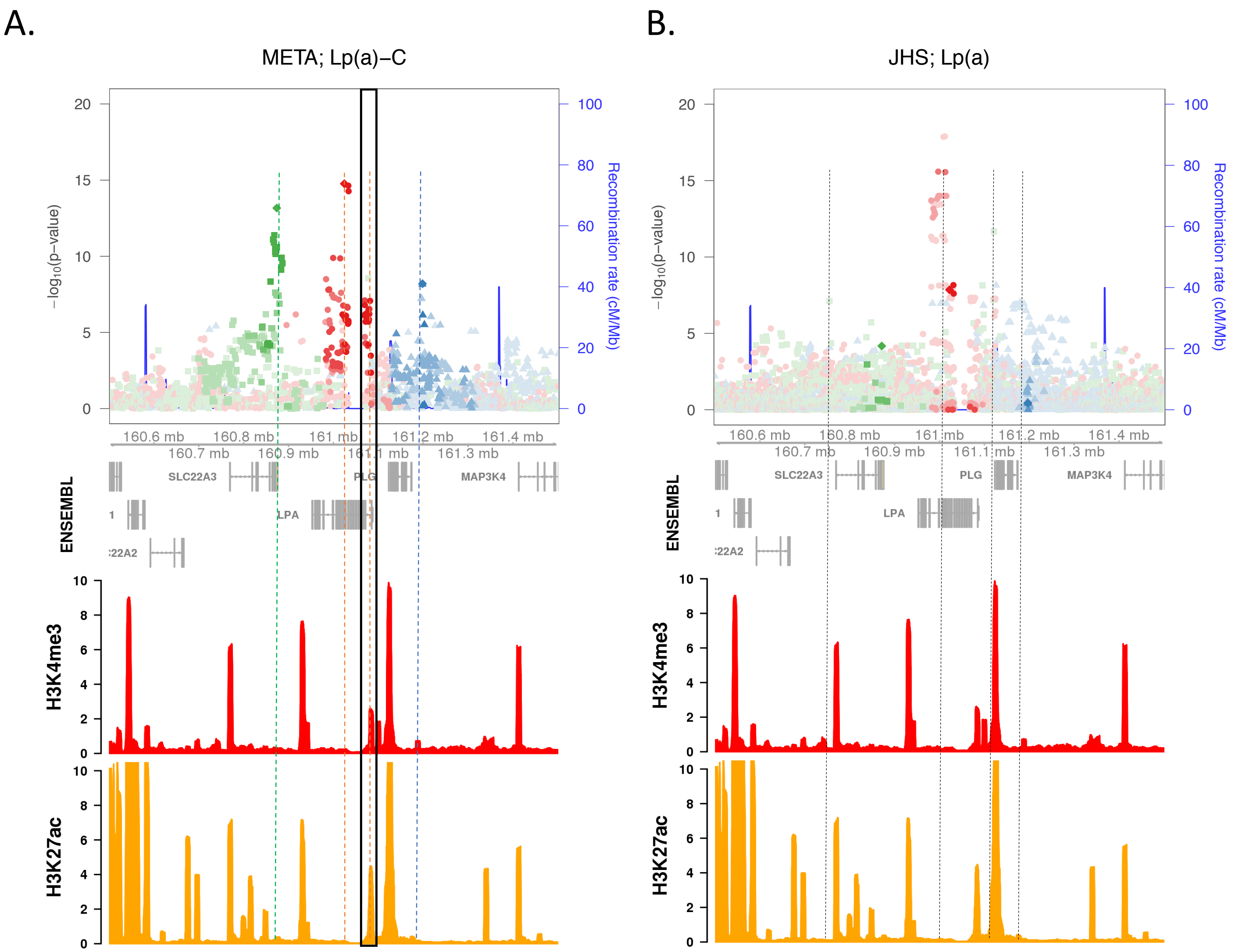

Supplementary Fig. 21 Overlap of Lp(a)-C and Lp(a) modifier variants with regulatory annotations from adult liver tissue in Roadmap (Roadmap cell type E066). The interaction p-values provided in the locus zoom plots (top panels) refer to those from an interaction model with KIV2-CN. Locus zoom plots of **A)** Lp(a)-C meta-analyzed modifier p-values between African Americans and Europeans, and **B)** Lp(a) modifier p-values in African Americans are shown, along with the following Roadmap adult liver regulatory annotations: H3K4me3 (indicative of promoter) and H3K27ac (indicative of enhancer) signal tracks, where all y-axes represent -log10(P) from consolidated histone signals in the following Roadmap website: http://egg2.wustl.edu/roadmap/data/byFileType/signal/consolidated/macs2signal/pval/. Vertical dotted lines are provided to aid in visualization of overlap between top modifier peaks in the locus zoom plot and regulatory annotations. The black rectangle in panel (A) highlights the top modifier peak in Estonians, where the top variant (rs4063600) is in strong LD ($r^2 = 0.88$) with the top meta-analyzed modifier variant in red noted in this figure (rs13192132). This is the only peak which clearly overlaps with significant ($P < 1e\text{-}2$) liver H3K4me3 and H3K27ac peaks. Further functional evidence is needed to understand whether this modifier variant impacts the effect of KIV2-CN on Lp(a)-C by acting as an enhancer or promoter of *LPA*.

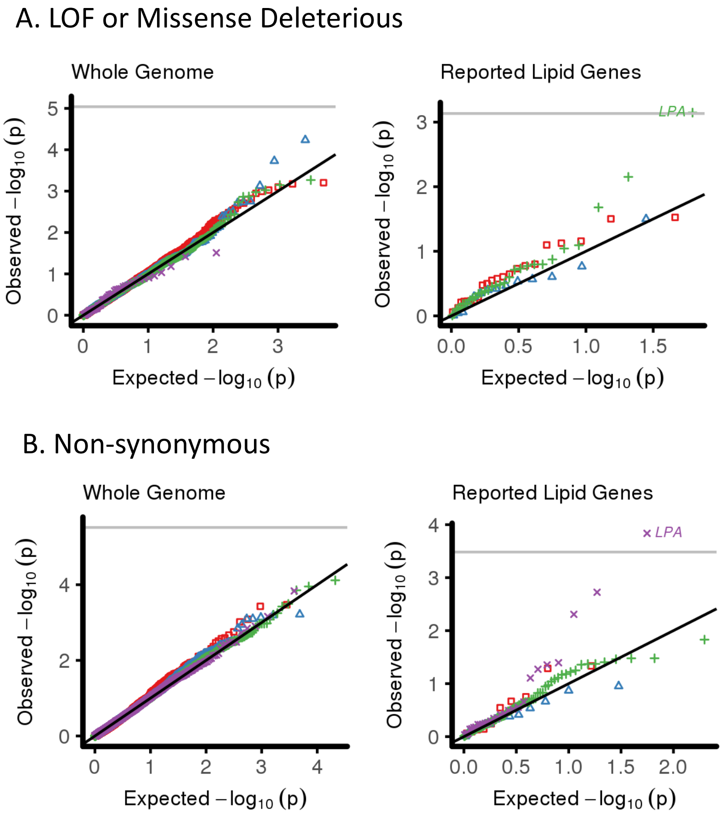

Supplementary Fig. 22 Quantile-quantile plots of meta-analyzed coding Lp(a)-C rare (MAF < 1%) variant association results across the whole genome and among reported lipid genes by cumulative MAF bin. **A)** Grouping together rare, LOF or missense deleterious (via the MetaSVM score) variants by gene. **B)** Grouping together rare, non-synonymous variants by gene. In all plots, the gray horizontal lines indicate the multiple-testing p-value of significance, with labeled genes indicating significant genes based on the number of genes tested. Dots are color coded by combined MAF bin (purple: MAF > 5%, green: MAF 1-5%, blue MAF 0.5-1%, red MAF 0.1-0.5%).

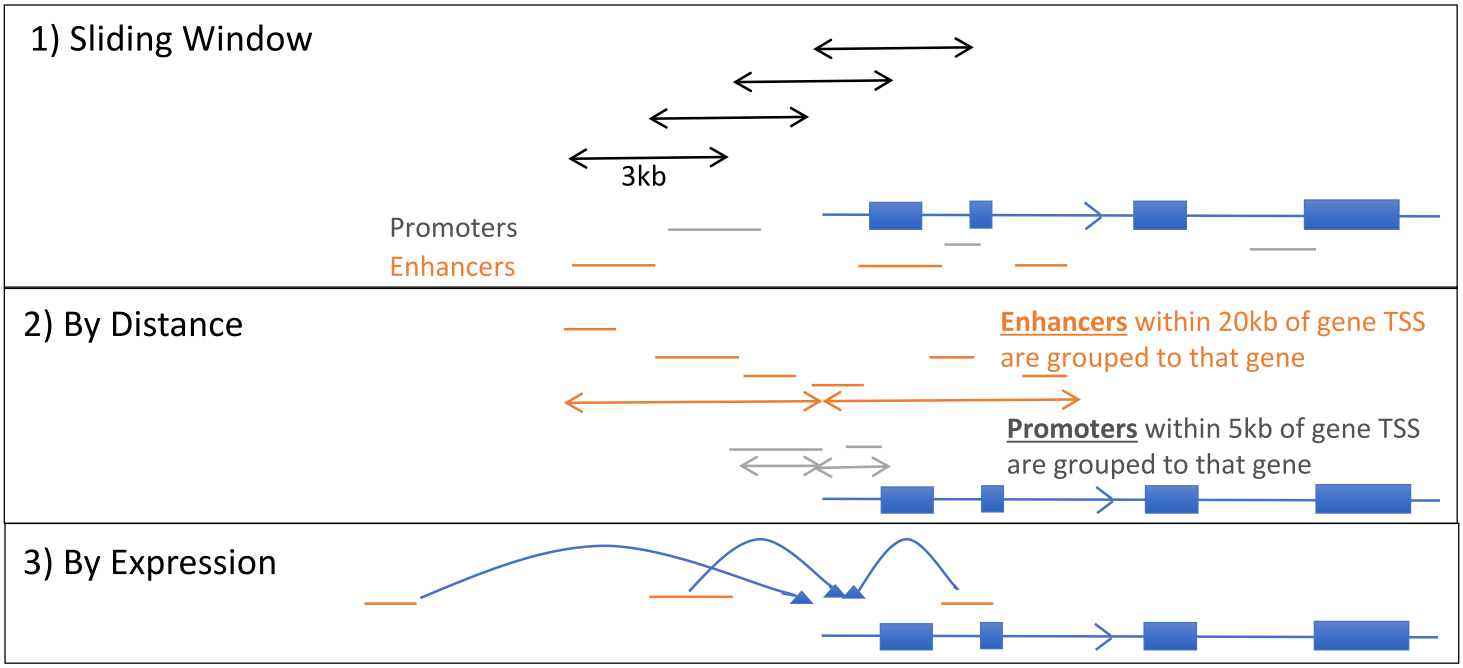

Supplementary Fig. 23 Schematic of non-coding rare variant association groupings. Three rare variant (MAF < 1%) grouping schemas were utilized in non-coding rare variant burden analyses: 1) Sliding Window: grouping together variants in liver enhancers or promoters from ChromHMM also overlapping strong DHS (DHS p-value < 1e-10) within 3kb windows overlapping by 1.5kb; 2) By Distance: grouping variants in ChromHMM liver enhancers within 20kb of the transcription start site (TSS) of a given gene to that gene, and also ChromHMM liver promoters within 5kb of the TSS of a given gene to that gene; 3) By Expression: grouping together enhancers in adult liver tissue to their predicted gene via a machine learning algorithm which uses the correlations of chromatin marks with gene expression across cell types.

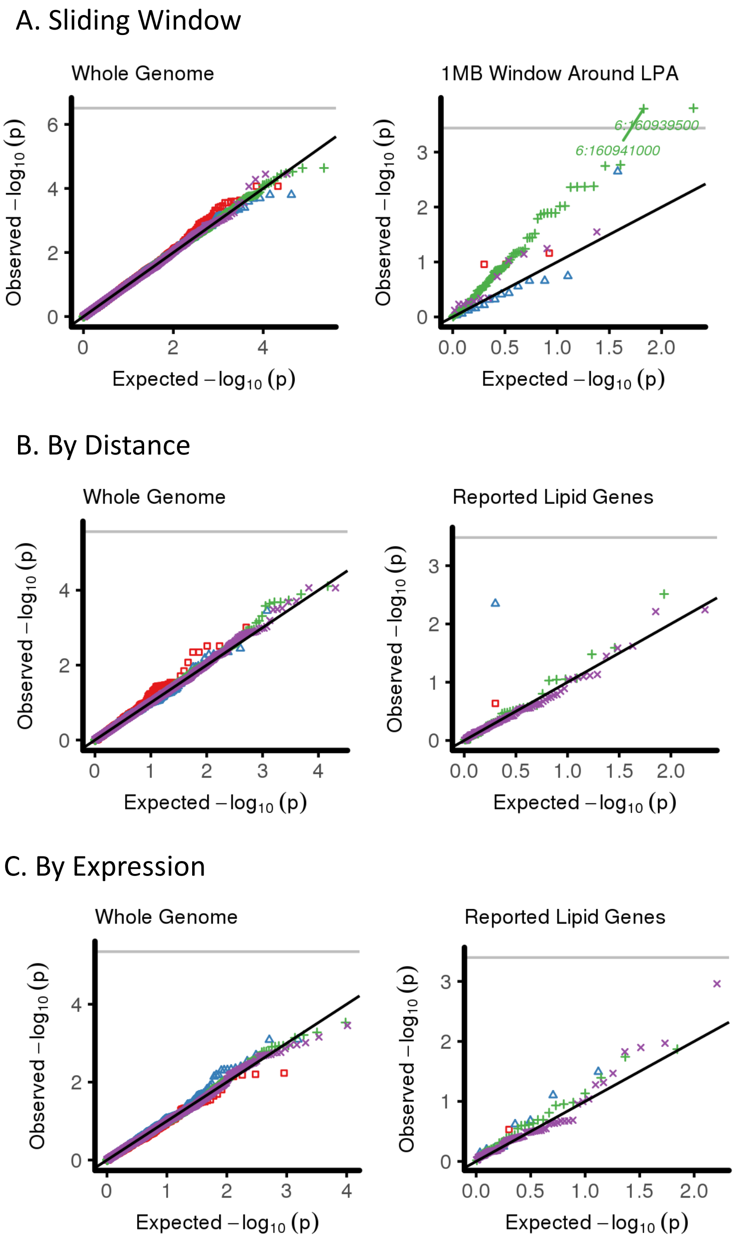

Supplementary Fig. 24 Quantile-quantile plots of meta-analyzed non-coding Lp(a)-C rare variant association results across the whole genome by cumulative MAF bin, after conditioning on KIV2-CN. **A)** Grouping together 3kb sliding windows overlapping by 1.5kb of are (MAF < 1%), variants overlapping liver enhancers or promoters in strong DHS (DHS p-value < 1x10^{-10}) gene. **B)** Grouping rare variants in strong DHS overlapping liver enhancers within 20kb of a gene transcription start site (TSS) and liver promoters within 5kb of a gene TSS to that gene. **C)** Grouping rare variants in liver enhancers to their predicted gene via a machine learning algorithm which uses the correlations of chromatin marks with gene expression across cell types. In all plots, the gray horizontal lines indicate the multiple-testing p-value of significance, with labeled genes indicating significant genes based on the number of genes tested. Dots are color coded by combined MAF bin (purple: MAF > 5%, green: MAF 1-5%, blue MAF 0.5-1%, red MAF 0.1-0.5%).

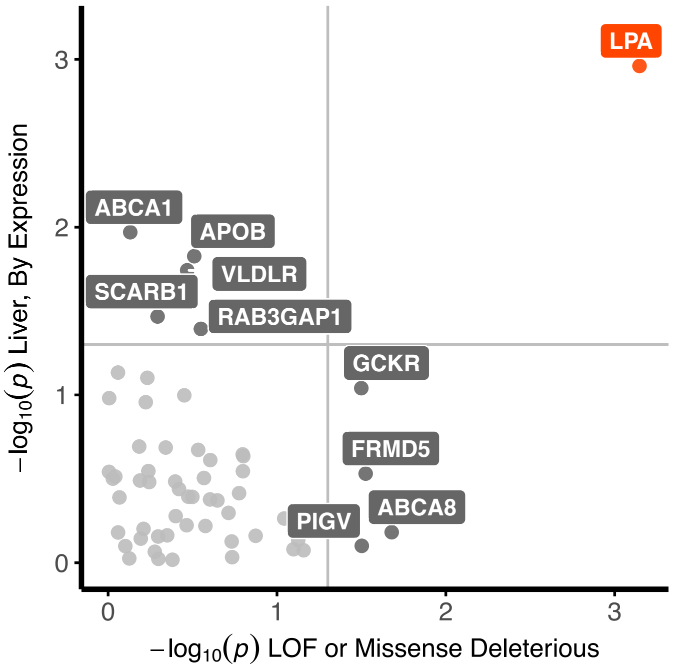

Supplementary Fig. 25 Rare variant association: comparing coding and non-coding gene-based burden associations among reported lipid genes. The Lp(a)-C SKAT association *P* values of rare variants (MAF < 1%) in coding sequence that are LOF or missense deleterious aggregated to their respective genes, and rare non-coding variants linked to gene by co-expression with liver, are compared against each other. Genes previously associated with lipids (listed in **Supplementary Data 15**) are shown, with *P* values representing meta-analyzed *P* across EST and JHS. Only *LPA* demonstrates nominal association ($P < 0.05$) for each of these tests. Lp(a)-C = lipoprotein(a) cholesterol; MAF = minor allele frequency; SKAT = Sequence Kernal Association Test

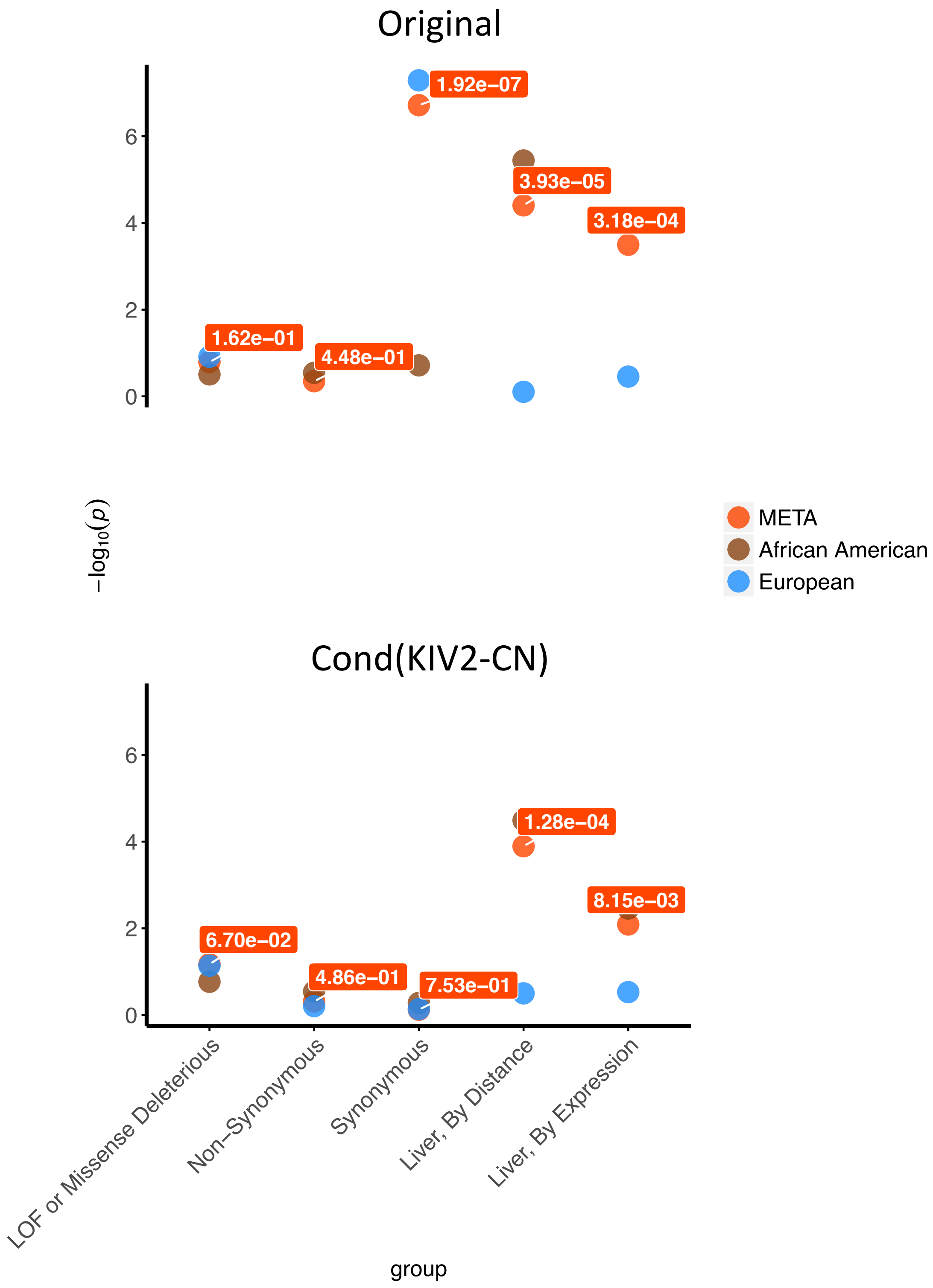

Supplementary Fig. 26 Comparison between Lp(a)-C RVAS results before and after conditioning on KIV2-CN for SLC22A3 by ethnicity and meta-analyzed. KIV2-CN = kringle IV-2 copy number; RVAS = rare variant association study

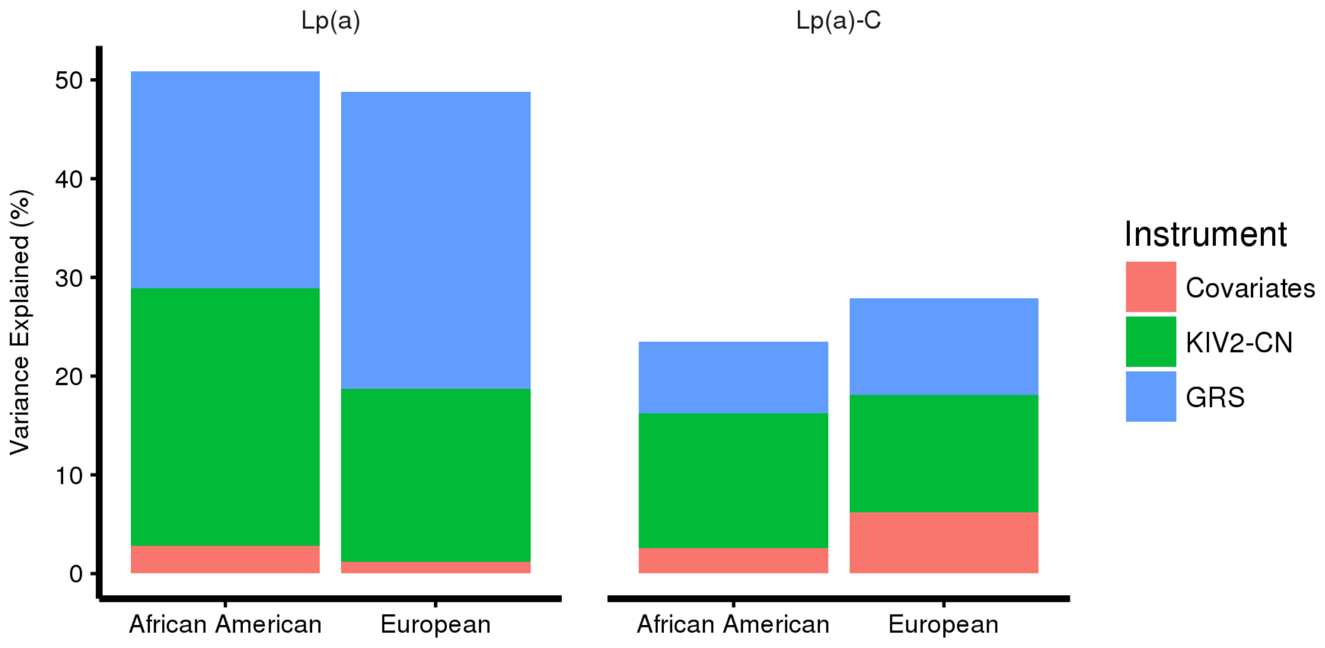

Supplementary Fig. 27 Variance explained for normalized Lp(a) and Lp(a)-C by ethnicity and instrumental variables. Cohorts included for Lp(a) include African Americans from WGSed JHS and Europeans from imputed FIN. Cohorts included for Lp(a)-C include African Americans from WGSed JHS and Europeans from WGSed EST. Variance explained from the following independent instrumental variables are included: (1) covariates included in single variant analysis (age, sex, fasting > 10h, sequencing batch, PC1-10); (2) a KIV2-CN score used in Mendelian randomization, which consists of directly genotyped KIV2-CN for JHS and EST and imputed KIV2-CN for FIN; and (3) a GRS comprised of betas of genetic variants after conditioning on KIV2-CN as used in Mendelian randomization. The exact values used to make this plot are provided in **Supplementary Table 10**. FIN = Finnish; GRS = genetic risk score; JHS = Jackson Heart Study; KIV2-CN = kringle IV-2 copy number; Lp(a)-C = lipoprotein(a) cholesterol; MESA = Multi-Ethnic Study of Atherosclerosis; OOA = Old Order Amish; PC = principal component; WGS = whole-genome sequenced

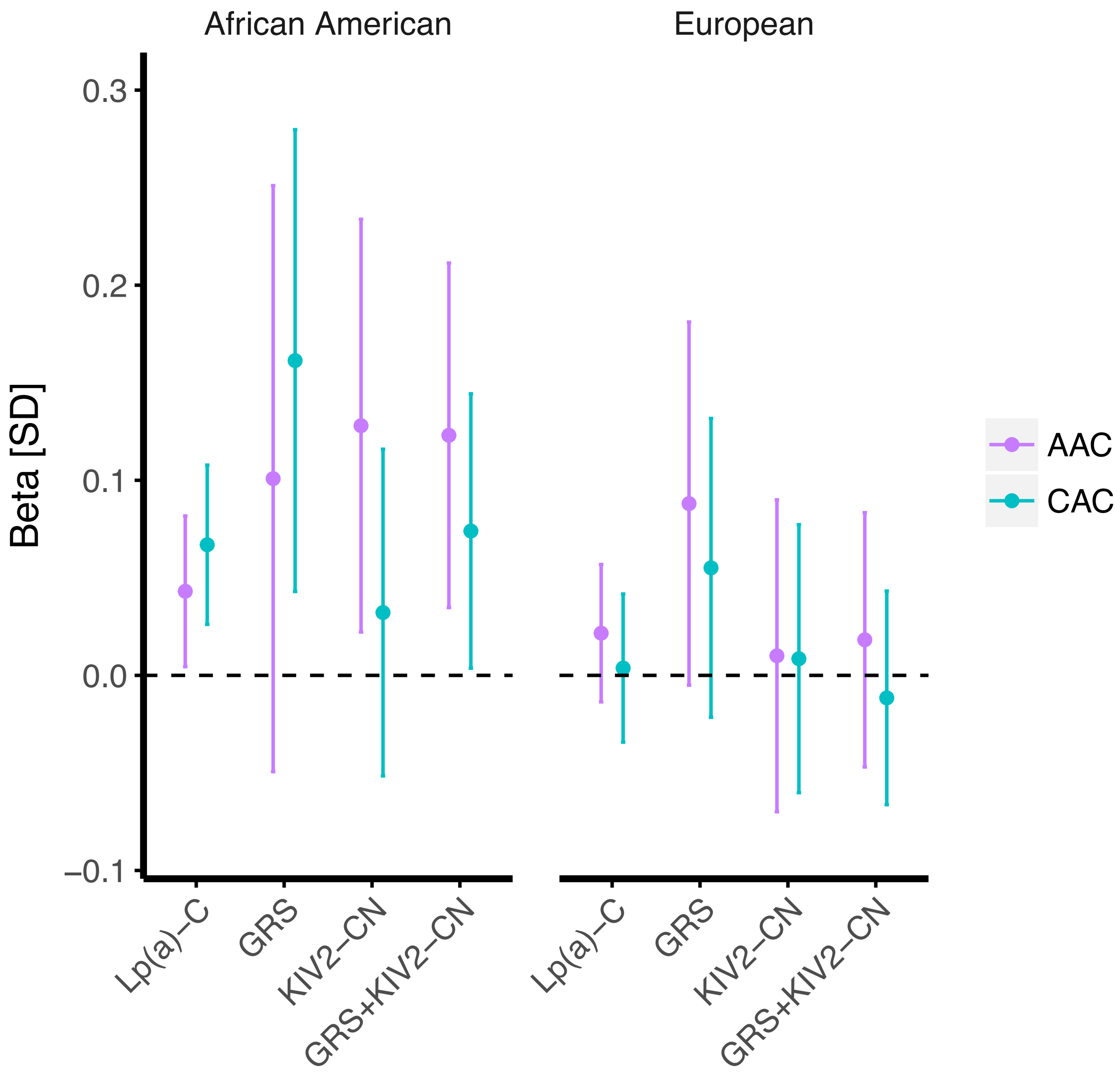

Supplementary Fig. 28 Association of *LPA* variant classes with subclinical measures. Mendelian randomization was performed using three genetic instruments: a weighted genetic risk score using variants conditioned on KIV2-CN at a 4Mb window around LPA (GRS), a KIV2-CN score, and a combined GRS+KIV2-CN score, and compared to the observational effects. The genetic instruments were all normalized such that 1 unit increase in the score is equal to 1SD increase in Lp(a)-C. Associations (Beta and 95% CI) of Lp(a)-C measurements and respective genetic instruments with standardized markers of subclinical atherosclerosis (CAC and AAC) among whole-genome sequences of African Americans from 1,701 JHS and 932 MESA participants, as well as Europeans from 1536 FHS, 1651 MESA, and 592 OOA individuals (exact values in **Supplementary Table 12**). Note: the African American GRS instruments were based off of the JHS GWAS and the European GRS instruments were based off of the FIN GWAS. AAC = Abdominal aortic calcium; CAC = coronary artery calcium; FHS = Framingham Heart Study; GRS = genetic risk score; GWAS = genome-wide association study; HR = hazard ratio; JHS = Jackson Heart Study; KIV2-CN = kringle IV-2 copy number; Lp(a)-C = lipoprotein(a) cholesterol; MESA = Multi-Ethnic Study of Atherosclerosis; OOA = Old Order Amish
