# Supplementary material for: Deep coverage whole genome sequences and plasma lipoprotein(a) in individuals of European and African ancestries
Source: Nat Commun. 2018 Jul 4;9:2606. doi: 10.1038/s41467-018-04668-w (PMC6031652; doi:10.1038/s41467-018-04668-w)
Supplement: Supplementary file 3 — Description of Additional Supplementary Files [file 41467_2018_4668_MOESM3_ESM.pdf]

Description of Additional Supplementary Files

File Name: Supplementary Data 1
Description: LASSO coefficients for 61 variants in KIV2-CN imputation model, ordered by their random forest importance.

File Name: Supplementary Data 2
Description: Single variant results for Lp(a)-C, conditioned on KIV2-CN, with metaanalyzed P < 1e-4.

File Name: Supplementary Data 3
Description: Single variant results for Lp(a), conditioned on KIV2-CN, with meta-analyzed P < 1e-4.

File Name: Supplementary Data 4
Description: Independent, genome-wide significant variants for Lp(a) by ethnicity.

File Name: Supplementary Data 5
Description: Genetic modifiers of KIV2-CN's effect on Lp(a)-C. A list of independent and bonferonni-corrected significant modifier variants (after clumping with r2 = 0.25) with AC > 20, by phenotype and cohort.

File Name: Supplementary Data 6
Description: LOF or MetaSVM missense RVAS: Coding rare variant association statistics for aggregates of rare (MAF<1%) LOF or MetaSVM-missense deleterious variants within a gene, conditioned on KIV2-CN.

File Name: Supplementary Data 7
Description: Non-synonymous RVAS: Coding rare variant association statistics for aggregates of rare (MAF<1%) non-synonymous within a gene, conditioned on KIV2-CN.

File Name: Supplementary Data 8
Description: Sliding window RVAS: Non-coding rare variant association statistics for aggregates of rare (MAF<1%) non-coding variants within 3kb sliding windows overlapping liver enhancers or promoters in strong DNAse hypersensitivity sites. Note: this analysis is not conditioned on KIV2-CN.

File Name: Supplementary Data 9
Description: Sliding window RVAS conditioned on KIV2-CN: Non-coding rare variant association statistics for aggregates of rare (MAF<1%) non-coding variants within 3kb sliding windows overlapping liver enhancers or promoters in strong DNAse hypersensitivity sites. Note: this analysis is conditioned on KIV2-CN.

File Name: Supplementary Data 10
Description: By-distance RVAS: Non-coding rare variant association statistics for aggregates of rare (MAF<1%) non-coding variants overlapping enhancers within 20kb and promoters within 5kb of gene transcription start sites in strong DNAse hypersensitivity sites. Note: this analysis is not conditioned on KIV2-CN.

File Name: Supplementary Data 11
Description: By-distance RVAS conditioned on KIV2-CN: Non-coding rare variant association statistics for aggregates of rare (MAF<1%) non-coding variants overlapping enhancers within 20kb and promoters within 5kb of gene transcription start sites in strong DNAse hypersensitivity sites. Note: this analysis is conditioned on KIV2-CN.

File Name: Supplementary Data 12
Description: By-expression RVAS: Non-coding rare variant association statistics for aggregates of rare (MAF<1%) non-coding enhancer variants linked to genes by gene expression in liver. Note: this analysis is not conditioned on KIV2-CN.

File Name: Supplementary Data 13
Description: By-expression RVAS conditioned on KIV2-CN: Non-coding rare variant association statistics for aggregates of rare (MAF<1%) non-coding enhancer variants linked to genes by gene expression in liver. Note: this analysis is conditioned on KIV2-CN.

File Name: Supplementary Data 14
Description: Variants used in the Lp(a)-C and Lp(a) genetic risk scores by cohort.

File Name: Supplementary Data 15
Description: Reported lipid loci from previous GWAS studies.
